# Postmastectomy Radiotherapy: An American Society of Clinical Oncology, American Society for Radiation Oncology, and Society of Surgical Oncology Focused Guideline Update

**DOI:** 10.1245/s10434-016-5558-8

**Published:** 2016-09-19

**Authors:** Abram Recht, Elizabeth A. Comen, Richard E. Fine, Gini F. Fleming, Patricia H. Hardenbergh, Alice Y. Ho, Clifford A. Hudis, E. Shelley Hwang, Jeffrey J. Kirshner, Monica Morrow, Kilian E. Salerno, George W. Sledge, Lawrence J. Solin, Patricia A. Spears, Timothy J. Whelan, Mark R. Somerfield, Stephen B. Edge

**Affiliations:** 1 Beth Israel Deaconess Medical Center, Boston, MA USA; 2 Memorial Sloan Kettering Cancer Center, New York, USA; 3 West Clinic Comprehensive Breast Center, Germantown, TN USA; 4 University of Chicago Medical Center, Chicago, IL USA; 5 Shaw Regional Cancer Center, Edwards, CO USA; 6 Duke University Medical Center, Durham, NC USA; 7 Hematology Oncology Associates of Central New York, East Syracuse, USA; 8 Roswell Park Cancer Institute, Buffalo, NY USA; 9 Stanford University Medical Center, Palo Alto, CA USA; 10 Albert Einstein Healthcare Network, Philadelphia, PA USA; 11 North Carolina State University, Raleigh, NC USA; 12 Juravinski Cancer Centre, McMaster University, Hamilton, Ontario Canada; 13 American Society of Clinical Oncology, 2318 Mill Road, Suite 800, Alexandria, VA 22314 USA

## Abstract

**Purpose:**

A joint American Society of Clinical Oncology, American Society for Radiation Oncology, and Society of Surgical Oncology panel convened to develop a focused update of the American Society of Clinical Oncology guideline concerning use of postmastectomy radiotherapy (PMRT).

**Methods:**

A recent systematic literature review by Cancer Care Ontario provided the primary evidentiary basis. The joint panel also reviewed targeted literature searches to identify new, potentially practice-changing data.

**Recommendations:**

The panel unanimously agreed that available evidence shows that PMRT reduces the risks of locoregional failure (LRF), any recurrence, and breast cancer mortality for patients with T1-2 breast cancer with one to three positive axillary nodes. However, some subsets of these patients are likely to have such a low risk of LRF that the absolute benefit of PMRT is outweighed by its potential toxicities. In addition, the acceptable ratio of benefit to toxicity varies among patients and physicians. Thus, the decision to recommend PMRT requires a great deal of clinical judgment. The panel agreed clinicians making such recommendations for individual patients should consider factors that may decrease the risk of LRF, attenuate the benefit of reduced breast cancer-specific mortality, and/or increase risk of complications resulting from PMRT. When clinicians and patients elect to omit axillary dissection after a positive sentinel node biopsy, the panel recommends that these patients receive PMRT only if there is already sufficient information to justify its use without needing to know additional axillary nodes are involved. Patients with axillary nodal involvement after neoadjuvant systemic therapy should receive PMRT. The panel recommends treatment generally be administered to both the internal mammary nodes and the supraclavicular-axillary apical nodes in addition to the chest wall or reconstructed breast.

**Electronic supplementary material:**

The online version of this article (doi:10.1245/s10434-016-5558-8) contains supplementary material, which is available to authorized users.

The American Society of Clinical Oncology (ASCO) guideline for the use of postmastectomy radiotherapy (PMRT) was published in 2001.^1^ This update of that guideline, completed in collaboration with the American Society for Radiation Oncology (ASTRO) and the Society of Surgical Oncology (SSO), focuses on key areas of ongoing controversy, including the use of PMRT for patients with one to three positive lymph nodes, use of PMRT for patients undergoing neoadjuvant systemic therapy (NAST), and selected technical aspects of PMRT, particularly the extent of regional nodal irradiation (RNI). The question of whether PMRT is indicated in women with T1-2 tumors and a positive sentinel node biopsy (SNB) who do not undergo completion axillary lymph node dissection (ALND) is also discussed.

The use of PMRT has been widely accepted for patients with four or more positive lymph nodes,^2^,^3^ but there is still controversy regarding the value of PMRT for those with one to three positive nodes. In 2014, the Early Breast Cancer Trialists’ Collaborative Group (EBCTCG) published an updated meta-analysis of the effects of PMRT.^4^ Its findings were summarized in a recent review by Cancer Care Ontario (CCO), which was reviewed by the panel (Table 1).^5^ There were 22 trials that in aggregate accrued 8,135 women between 1964 and 1986 who were randomly assigned to receive or not receive radiotherapy to the chest wall and regional lymph nodes after mastectomy and axillary surgery. Our panel focused on the results for the 3,786 women who underwent axillary dissection, defined as: inclusion in a protocol requiring at least an anatomic level I to II dissection, a median of 10 nodes examined in the study population, or individual patient data showing 10 or more recovered nodes. The EBCTCG performed additional subset analyses of those trials in which systemic therapy was routinely administered. Among the 1,133 patients with one to three positive nodes who had undergone axillary dissection and received systemic therapy, the 10-year rate of isolated LRF (defined as local recurrence without simultaneous or preceding distant failure) was 21.0 % without irradiation and 4.3 % with PMRT (*P* < .001). The 10-year rate for any recurrence (locoregional or distant) was 45.5 % without irradiation and 33.8 % with irradiation (*P* < .001), and the respective 20-year rates of breast cancer mortality were 49.4 % and 41.5 % (*P* = .01; relative risk, 0.78). There were no differences in the benefits of PMRT for patients with one positive node compared with those with two or three positive nodes with regard to any first recurrence or breast cancer mortality. However, the median follow-up for all patients in the meta-analysis was only 9.4 years, which means that relatively small numbers of patients were observable at 20 years.

**Table 1:** Results of the Fifth Cycle of the EBCTCG Review of the Role of PMRT

Nodal Status	No. of Patients	10-Year Local Recurrence Risk		20-Year Breast Cancer Mortality			20-Year Any-Cause Mortality		
		RT *v* no RT (%)	*P*	RT *v* no RT (%)	RR	*P*	RT *v* no RT (%)	RR	*P*
Mastectomy plus axillary dissection to ≥ level II (14 trials)									
Negative	700	3.0 *v* 1.6	>.1	28.8 *v* 26.6	1.18	>.1	47.6 *v* 41.6	1.23	.03
Positive	3,131	8.1 *v* 26.0	<.001	58.3 *v* 66.4	0.84	.001	65.4 *v* 70.4	0.89	.01
One to three positive	1,314	3.8 *v* 20.3	<.001	42.3 *v* 50.2	0.80	.01	53.5 *v* 56.5	0.89	>.1
One to three positive plus systemic therapy	1,133	4.3 *v* 21.0	<.001	41.5 *v* 49.4	0.78	.01	52.6 *v* 55.5	0.86	.08
≥ Four positive nodes	1,772	13.0 *v* 32.1	<.001	70.7 *v* 80.0	0.87	.04	75.1 *v* 82.7	0.89	.05
≥ Four positive nodes plus systemic therapy	1,677	13.6 *v* 31.5	<.001	70.0 *v* 78.0	0.89	.08	74.9 *v* 82.0	0.90	>.1
Mastectomy plus axillary sampling (nine trials)									
Negative	870	3.7 *v* 17.8	<.001	32.0 *v* 35.8	0.97	>.1	46.1 *v* 49.9	1.00	>.1
Positive	2,541	6.3 *v* 37.2	<.001	55.6 *v* 68.2	0.74	<.001	63.1 *v* 71.8	0.79	<.001
Mastectomy only (four trials)									
Clinically negative	2,896	16.1 *v* 35.4	<.001	50.8 *v* 53.1	0.97	>.1	62.8 *v* 61.8	1.06	>.1
Clinically positive	1,481	18.0 *v* 45.0	<.001	56.6 *v* 63.3	0.86	.03	67.1 *v* 71.5	0.91	>.1

NOTE. Data adapted with permission^5^

Abbreviations: EBCTCG, Early Breast Cancer Trialists’ Collaborative Group; PMRT, postmastectomy radiotherapy; RR, relative risk; RT, radiotherapy

The most recent EBCTCG meta-analysis provides evidence that PMRT is highly effective at preventing LRF and, in an era of intermediate to high risk for recurrence, reduces the risk of patients with one to three nodes developing distant metastases and dying as a result of breast cancer. However, more recent evidence suggests that these findings may not be directly applicable to all patients with one to three positive nodes in the current era, when many of these patients are at much lower risk for recurrence. The trials included by the EBCTCG were predominantly conducted in the 1970s and 1980s. Rates of LRF and any recurrence reported in this meta-analysis were considerably higher than those reported in many contemporaneous and later series. Multiple studies from North America, Europe, and Asia of patients treated with mastectomy and systemic therapy without irradiation since 1990 have reported much lower 5- to 10-year actuarial LRF rates, with the most recent series usually reporting local LRF rates lower than 10 % (Table 2). The divergence between the rates reported by the EBCTCG and contemporary series seems to have increased over time since the original ASCO PMRT guideline was published.

**Table 2:** LRF Rates Without RT After Modified Radical Mastectomy and Chemotherapy (with or without endocrine therapy) in Modern Series of Patients With pT1-2N1 Breast Cancer With Median Follow-Up of ≥5 Years

Institution	Accrual Dates	No. of Patients	Median Follow-Up (months)	Measure	Rate (%)
MDACC^6^	1975–1994	466	116	10-year actuarial	14
ECOG^7^	1978–1987	1,018	145	10-year actuarial	13
NSABP^8^	1984–1994	2,957	133	10-year actuarial	13
BCCA^9^	1989–1997	821	92	10-year actuarial	17
Ankara, Turkey^10^	1990–2004	326	70	Crude	4
MGH^11^	1990–2004	165	84	10-year actuarial	11
Shikoku, Japan^12^	1990–2002	248	82	8-year actuarial	5*
Kaohsiung, Taiwan^13^	1990–2008	155	102	10-year actuarial	11^†^
Seoul, Korea^14^	1992–2004	401	68	10-year actuarial	20^†^
CALGB 9344^15^	1994–1997	254	67	Crude	8
MSKCC^16^	1995–2006	924	84	5-year actuarial	4^†^
Tampa, FL^17^	1996–2007	204	66	Crude	10
EIO^18^	1997–2001	262	120	10-year actuarial	10*
MDACC^19^	1997–2002	266	90	10-year actuarial	4
Cleveland Clinic^20^	2000–2007	271	62	5-year actuarial	9^†^
MDACC^21^	2000–2007	385	84	10-year actuarial	7
Tianjin, China^22^	2001–2005	368	86	8-year actuarial	11

NOTE. Rates include patients with both isolated LRF and simultaneous LRF and distant metastases, unless otherwise noted

Abbreviations: BCCA, British Columbia Cancer Agency; CALGB, Cancer and Leukemia Group B; ECOG, Eastern Cooperative Oncology Group; EIO, European Institute of Oncology; LRF, locoregional failure; MDACC, MD Anderson Cancer Center; MGH, Massachusetts General Hospital; MSKCC, Memorial Sloan Kettering Cancer Center; NSABP, National Surgical Adjuvant Breast and Bowel Project; RT, radiotherapy

* Isolated locoregional recurrences only

^†^Not stated whether isolated or total locoregional recurrence rate reported

This trend of decreasing LRF rates over time likely results from multiple factors. These include decreasing average tumor sizes and a smaller average number of positive lymph nodes than that reported in some of the earlier randomized trials of PMRT^23^; a higher average number of resected lymph nodes in axillary lymph node dissections in more recent years, reflecting more complete axillary clearance; and the use of increasingly effective systemic therapy regimens. The trials included in the EBCTCG meta-analysis were primarily conducted in the 1970s and 1980s, with a median of fewer than 10 resected lymph nodes. Chemotherapy was most commonly cyclophosphamide, methotrexate, and fluorouracil; methotrexate plus fluorouracil; or single-agent cyclophosphamide or melphalan (Data Supplement Table 1).^4^ Only a few trials included early doxorubicin-containing regimens. Ovarian irradiation was used in three trials. Tamoxifen was often administered for short courses (eg, for only 1 year in the Danish trial for postmenopausal women^24^), and generally, patients did not receive both tamoxifen and chemotherapy. The results of the trials included in the meta-analysis thus do not reflect the advances in systemic therapy made since 1986 and hence are not representative of current practice. These advances include the advent of adjuvant use of taxanes, dose-dense chemotherapy schedules, supportive care measures that improve chemotherapy adherence, adjuvant trastuzumab and other human epidermal growth factor receptor 2-targeted drugs for patients with human epidermal growth factor receptor 2-positive cancers, adjuvant aromatase inhibitor therapy for postmenopausal women, combined endocrine blockade (ovarian ablation plus aromatase inhibitor therapy) in premenopausal women, and the use of prolonged adjuvant hormonal therapy (eg, 10 years of tamoxifen or 5 years of tamoxifen followed by 5 years of aromatase inhibitor therapy). As adjuvant systemic therapy improves, the risk of LRF is likely to decrease, and hence, the benefits seen of PMRT might decrease in both relative and absolute terms.

In view of the importance of the question of whether the benefits of PMRT (including its impact on overall survival) outweigh its known toxicities for this large group of patients with cancer, ASCO convened a guideline update panel in collaboration with ASTRO and SSO to provide recommendations for the use of PMRT in patients with T1 and T2 tumors (≤5 cm) and one to three involved nodes. On the basis of its discussion of the current concerns of clinicians and recent publications,^25^–^27^ and with the approval of the ASCO Breast Cancer Guideline Advisory Group co-chairs, the panel also addressed recommendations regarding the use of PMRT for patients undergoing SNB without ALND and for those treated with NAST and the optimal extent of RNI.

## Focused Guideline Update Questions

Question 1: Is PMRT indicated in patients with T1-2 tumors with one to three positive axillary lymph nodes who undergo ALND?

Question 2: Is PMRT indicated in patients with T1-2 tumors and a positive SNB who do not undergo completion ALND?

Question 3: Is PMRT indicated in patients presenting with clinical stage I or II cancers who have received NAST?

Question 4: Should RNI include the internal mammary (IMNs) and/or supraclavicular-axillary apical nodes when PMRT is used in patients with T1-2 tumors with one to three positive axillary nodes?

## Methods

### Guideline Update Process

ASCO uses a signals approach to facilitate guideline updating. This approach is intended to identify new, potentially practice-changing data that might translate into revised practice recommendations. The approach relies on targeted literature searching and the expertise of ASCO guideline panel members to identify signals. The Methodology Supplement published with this article provides additional information about the signals approach.

The 2014 publication of the EBCTCG meta-analysis^4^ provided the signal for this focused update. Based in large part on this signal, the ASCO Breast Cancer Advisory Group ranked updating the ASCO PMRT guideline question concerning use of PMRT for patients with one to three positive lymph nodes as a high priority. To that end, a joint ASCO–ASTRO–SSO panel was formed to review the evidence and formulate updated recommendations for practice.

The systematic review of literature by the CCO of locoregional therapy for locally advanced breast cancer guideline provided the primary evidentiary basis for the ASCO guideline focused update.^5^ The CCO literature searches identified systematic reviews, meta-analyses, randomized controlled trials, cohort studies, and clinical practice guidelines that studied locoregional therapy for locally advanced breast cancer. For studies to be included in the analysis, the CCO required them to have at least 50 patients, have a prospective design, and provide a statistical comparison of the interventions of interest. At the request of ASCO, CCO guideline staff conducted an updated search of the CCO systematic review. The yield from the updated CCO search was reviewed for new, potentially practice-changing data.

Two additional targeted searches were conducted by the ASCO Guidelines Division staff to identify systematic reviews, meta-analyses, and randomized controlled trials of PMRT in women who had received neoadjuvant chemotherapy and of technical aspects of PMRT, especially RNI. A third targeted literature search and review was conducted to identify single-center and multi-institutional prospective and retrospective studies of patients treated since the PMRT trials in the EBCTCG meta-analysis were completed. Inclusion criteria for this targeted review were: retrospective or prospective study published between January 2001 and July 2015, patients accrued from 1985 or later, 150 or more patients explicitly identified with T1-2 cancers with one to three positive nodes, patients not treated with neoadjuvant chemotherapy, and median follow-up 48 months or longer.

The entire panel contributed to the development of the guideline, provided critical review, and finalized the guideline recommendation. All ASCO guidelines are reviewed and approved by the ASCO Clinical Practice Guidelines Committee. This focused update was reviewed by the ASTRO Guidelines Committee and approved by the ASTRO Board of Directors; the update was also reviewed by the SSO Breast Cancer Disease Site Work Group and approved by the SSO Quality Committee and Executive Council.

### Guideline Disclaimer

The clinical practice guideline and other guidance published herein are provided by ASCO to assist providers in clinical decision making. The information herein should not be relied upon as being complete or accurate, nor should it be considered as inclusive of all proper treatments or methods of care or as a statement of the standard of care. With the rapid development of scientific knowledge, new evidence may emerge between the time information is developed and when it is published or read. The information is not continually updated and may not reflect the most recent evidence. The information addresses only the topics specifically identified herein and is not applicable to other interventions, diseases, or stages of disease. This information does not mandate any particular course of medical care. Furthermore, the information is not intended to substitute for the independent professional judgment of the treating provider, because the information does not account for individual variation among patients. Recommendations reflect high, moderate, or low confidence that the recommendation reflects the net effect of a given course of action. The use of words like “must,” “must not,” “should,” and “should not” indicates that a course of action is recommended or not recommended for either most or many patients, but there is latitude for the treating physician to select other courses of action in individual cases. In all cases, the selected course of action should be considered by the treating provider in the context of treating the individual patient. Use of the information is voluntary. ASCO provides this information on an as-is basis and makes no warranty, express or implied, regarding the information. ASCO specifically disclaims any warranties of merchantability or fitness for a particular use or purpose. ASCO assumes no responsibility for any injury or damage to persons or property arising out of or related to any use of this information or for any errors or omissions.

This guideline reflects the most recent information as of the submission date. For the most recent information or to submit new evidence, please visit www.asco.org/pmrt-guideline and the ASCO Guidelines Wiki (www.asco.org/guidelineswiki).

### Guideline and Conflicts of Interest

The expert panel was assembled in accordance with the ASCO Conflict of Interest Management Procedures for Clinical Practice Guidelines summarized at www.asco.org/rwc). Members of the panel completed the ASCO disclosure form, which requires disclosure of financial and other interests that are relevant to the subject matter of the guideline, including relationships with commercial entities that are reasonably likely to experience direct regulatory or commercial impact as a result of promulgation of the guideline. Categories for disclosure include: employment; leadership; stock or other ownership; honoraria, consulting or advisory role; speaker’s bureau; research funding; patents, royalties, other intellectual property; expert testimony; travel, accommodations, expenses; and other relationships. In accordance with these procedures, the majority of the members of the panel did not disclose any such relationships.

## Recommendations

### Clinical Question 1

Is PMRT indicated in patients With T1-2 tumors with one to three positive axillary lymph nodes who undergo ALND?

#### Updated Recommendations

##### Recommendation 1a

The panel unanimously agreed that the available evidence shows that PMRT reduces the risks of LRF, any recurrence, and breast cancer mortality for patients with T1-2 breast cancer with one to three positive axillary nodes (type: evidence based; evidence quality: high; strength of recommendation: strong). However, some subsets of these patients are likely to have such a low risk of LRF that the absolute benefit of PMRT is outweighed by its potential toxicities (type: evidence based; evidence quality: intermediate; strength of recommendation: strong). In addition, the acceptable ratio of benefit to toxicity varies among patients and physicians. Thus, the decision to recommend PMRT or not requires a great deal of clinical judgment. The panel agreed clinicians making such recommendations for individual patients should consider factors that may decrease the risk of LRF, attenuate the benefit of reduced breast cancer-specific mortality, and/or increase the risk of complications resulting from PMRT. These factors include: patient characteristics (eg, age >40–45 years, limited life expectancy because of older age or comorbidities, or coexisting conditions that might increase the risk of complications), pathologic findings associated with a lower tumor burden (eg, T1 tumor size, absence of lymphovascular invasion, presence of only a single positive node and/or small size of nodal metastases, or substantial response to NAST), and biologic characteristics of the cancer associated with better outcomes and survival and/or greater effectiveness of systemic therapy (eg, low tumor grade or strong hormonal sensitivity; type: informal consensus; evidence quality: intermediate; strength of recommendation: moderate). There are several risk-adaptive models that physicians may find useful in explaining the benefits of PMRT during shared decision making with patients. However, the panel found insufficient evidence to endorse any specific model or to unambiguously define specific patient subgroups to which PMRT should not be administered (type: no recommendation; evidence quality: low; strength of recommendation: weak). Further research is needed on how to accurately estimate individuals’ risk of LRF and hence their potential reductions in LRF and breast cancer mortality.

##### Recommendation 1b

The decision to use PMRT should be made in a multidisciplinary fashion through discussion among providers from all treating disciplines early in a patient’s treatment course (soon after surgery or before or soon after the initiation of systemic therapy), either in the context of a formal tumor board or by referral (type: informal consensus; evidence quality: insufficient; strength of recommendation: strong).

##### Recommendation 1c

Decision making must fully involve the patient, whose values as to what constitutes sufficient benefit and how to weigh the risk of complications against this in light of the best information the treating physicians can provide regarding PMRT in her situation must be respected and incorporated into the final treatment choice (type: informal consensus; evidence quality: insufficient; strength of recommendation: strong).

#### Literature Review and Analysis

The grouping of patients with breast cancer in relation to the number of involved axillary nodes (eg, zero, one to three, four to nine, or >10) is of long standing in clinical trials and has been codified in the American Joint Committee on Cancer and Union for International Cancer Control TNM staging systems. However, these divisions have arguably been made much less important by improved understanding of the biology of breast cancer. Certainly, there is likely little difference in prognosis or benefit of therapy for women with three versus four positive nodes, but there may well be substantial difference between those with a single node with minimal metastatic burden and those with three nodes with bulky metastases. In addition, the prognostic and therapeutic impacts of a particular number of positive nodes may be different in patients who undergo SNB without ALND than in those who undergo ALND, because the total number of positive nodes may only be inferred if only SNB is performed (Clinical Question 2 provides a discussion of the use of PMRT for patients treated with SNB without completion ALND). Nonetheless, although such division of patients into groups on the basis of the number of involved nodes may be less operationally useful than in the past, it remains deeply embedded within the structure of clinical trial design, data analysis, and staging, making it difficult to avoid addressing the issue of the value of PMRT without using this categorization. Therefore, the panel focused its attention on patients with one to three positive nodes while recognizing that distinctions between the historic nodal prognostic groups are increasingly difficult to justify.

The recent EBCTCG meta-analysis provides considerable evidence that for patients with T1-2 tumors with one to three nodes PMRT reduces the risk of developing any recurrence and dying as a result of breast cancer and markedly reduces the risk LRF. However, as previously noted, the LRF rate in patients not undergoing irradiation in the trials reported in the EBCTGC (21 %) was higher than that seen in most studies using more modern surgery and more contemporary adjuvant systemic therapies (most studies report LRF rates ranging from 4 % to 10 %; Table 2), leading the panel to question the generalizability of the EBCTCG results to all patients. Hence, not all patients treated with current standard axillary dissection and modern systemic therapy regimens will likely benefit sufficiently from PMRT to justify its use. Although morbidities resulting from PMRT have diminished over time because of improved radiation treatment planning and delivery techniques, compared with those used at the time of the trials reported in the EBTCG meta-analysis,^28^ they are not negligible. Some, such as radiation-induced cardiac disease^29^–^32^ and cancers,^33^ may take decades to appear; hence, their ultimate rates with modern PMRT techniques cannot yet be ascertained. RNI may increase the risk of lymphedema, especially in patients who also undergo ALND.^26^,^34^ Furthermore, many more patients now undergo breast reconstructive surgery. Administration of PMRT can worsen cosmetic results and increase the risk of both short- and long-term complications.^35^,^36^ Therefore, if it can be determined that the risk of LRF is low for certain subgroups of patients, then by inference any reduction in breast cancer mortality would be low or negligible, making PMRT unadvisable in such patients.

Previous estimates of the value of irradiation in patients treated with breast-conserving surgery have suggested that radiation treatment has reduced impact on survival when it changes the risk of local recurrence risk by less than 10 %.^37^ Comparable data are not available for patients with one to three positive nodes in the EBCTCG meta-analysis, although some studies support that smaller reductions in regional recurrence can reduce breast cancer mortality (as discussed in Clinical Question 4). Furthermore, individual clinicians and patients will differ on the precise level of benefit (either a reduction in LRF, any recurrence, or breast cancer mortality) they feel is sufficient to justify PMRT, even if its impact could be accurately calculated. In addition, some have argued that the total risk of relapse would be a better surrogate or proxy than LRF to guide this decision making, because PMRT may eradicate areas of disease not destroyed by systemic therapy that could result in eventual tumor dissemination but may not manifest themselves clinically at those sites before (or after) systemic relapse. Hence, the panel cannot set a specific threshold for a risk of LRF that would justify the use or omission of PMRT.

Because the absolute benefit of PMRT seems likely to be greater for those patients with a higher risk of recurrence, the panel supports the use of a risk-adaptive strategy to guide patient selection for PMRT that attempts to balance the potential benefits of PMRT against its potential harms. Such a calculation should be based on: patient characteristics predicting for a lower risk of LRF or shorter life expectancy or an increased risk of complications, pathologic findings predicting a smaller tumor burden after definitive surgery, biologic factors predicting better outcome and survival, and the expected effectiveness of planned systemic therapy in order to balance benefits against harms. Evidence regarding the importance of individual factors is often based on older studies and limited data and is often contradictory. The effect of these factors in different studies often varies substantially. Until more definitive evidence is available, using its best clinical opinion, the panel recommends that such a strategy or risk estimation include assessment of patient age as it affects the risk of LRF,^7^,^10^,^21^,^22^,^38^,^39^ estimated life expectancy in relation to age and comorbid conditions that might reduce life expectancy^40^–^44^ or increase the risk of complications,^45^–^49^ tumor size,^6^,^7^ axillary lymph node burden (number of positive nodes,^9^,^16^,^19^,^22^ nodal ratio,^10^,^20^,^22^,^38^ and size of nodal tumor deposits^16^,^17^,^19^), tumor grade,^11^,^16^,^20^,^22^,^38^,^50^ lymphovascular invasion,^10^,^16^,^21^,^22^,^38^,^51^ biomarker or receptor status,^7^,^8^,^16^,^22^,^38^,^52^–^56^ and planned systemic therapy (Data Supplement provides discussions of these and additional factors, such as margin status^57^,^58^ and extranodal extension^16^,^19^,^20^). Several groups have proposed prognostic models to estimate the risk of LRF after mastectomy by combining several of these factors.^10^,^16^,^20^,^22^,^38^ Although the panel cannot endorse any specific model, because the models have yet to be validated, physicians may find them useful in explaining the benefits of PMRT. Further research is needed on these models and how to accurately estimate an individual’s risk of LRF and hence the potential reduction in LRF and breast cancer mortality.

Finally, the panel noted that the United Kingdom Medical Research Council SUPREMO (Selective Use of Postoperative Radiotherapy After Mastectomy) trial^59^ randomly allocated approximately 1,600 patients with high-risk node-negative disease and one to three positive nodes (including when found after neoadjuvant chemotherapy) from June 2006 to April 2013 to receive PMRT or not. The results of this trial may eventually help determine which patients are most likely to benefit from PMRT when modern systemic therapy and surgery are used.

### Clinical Question 2

Is PMRT indicated in patients with T1-2 tumors and a positive SNB who do not undergo completion ALND?

#### Recommendation

For patients with clinical T1-2 tumors with clinically negative nodes, SNB is now generally performed at the time of mastectomy, with omission of ALND if the nodes are negative. ALND has generally been performed if the nodes are positive, but there is increasing controversy about whether this is always necessary, especially if there is limited disease in the affected nodes. The panel recognizes that some clinicians omit axillary dissection with one or two positive sentinel nodes in patients treated with mastectomy. This practice is primarily based on extrapolation of data from randomized trials of patients treated exclusively or predominantly with breast-conserving surgery and whole-breast irradiation or breast plus axillary irradiation. In such cases where clinicians and patients elect to omit axillary dissection, the panel recommends that these patients receive PMRT only if there is already sufficient information to justify its use without needing to know that additional axillary nodes are involved (type: informal consensus; evidence quality: weak; strength of recommendation: moderate).

#### Literature Review and Analysis

The discussion of Clinical Question 1 was based on the assumption that patients had undergone level I to II ALND. However, it is not clear whether the clinical implications of positive nodes found on SNB are the same as those for patients undergoing ALND, because the extent of surgery is smaller, and there is a substantial chance of additional positive nonsentinel nodes remaining in the patient treated with SNB alone. The 2014 ASCO Panel on Sentinel Lymph Node Biopsy discussed the role of ALND for patients undergoing mastectomy (in Clinical Question 2.2 of its guideline).^60^ It concluded the following: “Clinicians may offer ALND for women with early-stage breast cancer with nodal metastases found on SNB who will undergo mastectomy. Type: evidence based; benefits outweigh harms. Evidence quality: low. Strength of recommendation: weak.” However, that panel did not discuss whether this applied equally to patients who are or are not likely to receive PMRT.

Some clinicians question the need for ALND after mastectomy in women based on extrapolation of the findings from three trials that randomly assigned patients with positive sentinel nodes to undergo ALND or no further axillary surgery. The ACOSOG (American College of Surgeons Oncology Group) Z0011 trial included 856 patients with one or two sentinel node micro- or macrometastases undergoing breast-conserving therapy, including whole-breast irradiation.^61^,^62^ At a median follow-up of 6.3 years, there was no difference between patients allocated to ALND or no ALND with regard to locoregional recurrence or survival. A small percentage of patients underwent axillary irradiation in violation of the protocol, but the effect of this on outcome is not known.^63^ The IBCSG (International Breast Cancer Study Group) 23-01 trial included 931 women with one or two sentinel node micrometastases; those with macrometastases were excluded.^64^ Similar to the ACOSOG Z0011 trial, patients were randomly assigned to undergo SNB alone or completion ALND. Patients who had undergone mastectomy were eligible and constituted 9 % of the study population (84 patients). Breast irradiation without nodal irradiation was administered to 81 % of those treated with breast-conserving surgery, but PMRT was not administered to those who had undergone mastectomy. This trial also showed no difference in rate of regional or distant failure between the arms at a median follow-up of 5 years. Of note, there were no regional nodal recurrences in 42 patients treated with mastectomy who did not receive PMRT or ALND. Finally, the EORTC (European Organisation for Research and Treatment of Cancer) 0981-22023 trial AMAROS [After Mapping of the Axilla, Radiotherapy or Surgery]) compared ALND with breast plus axillary irradiation in 1,525 women with one or two sentinel node micro- or macrometastases.^65^ Most had undergone breast-conserving surgery and whole-breast irradiation, but 18 % of the participants had undergone mastectomy, of whom approximately one third had also received chest wall irradiation. There were no significant differences in any measure of recurrence or mortality at a median follow-up of 6.1 years. Rates of nodal failure in the patients who had undergone mastectomy were not separately reported for this trial. However, in both studies, the total number of women treated with mastectomy was small.

Hence, at present, some physicians feel that ALND can be omitted for patients undergoing mastectomy who have similar findings on SNB to those of patients eligible for the randomized trials, particularly if PMRT is administered, whereas others feel ALND should still be performed. Although there are insufficient data to support or refute these opinions, the panel agreed that it is inappropriate to subject patients to the potential acute and long-term toxicities of PMRT (including rare but potentially fatal second cancers and cardiac events) without careful consideration of whether these are justified compared with the potential toxicities of ALND. The decision as to how to integrate ALND and PMRT for an individual patient should be a multidisciplinary effort that considers the treatment program as a whole. Thus, PMRT should be administered if there is otherwise sufficient evidence to warrant its use when ALND is omitted and the potential toxicities of PMRT are felt to be justified, and ALND should be used when the totality of the evidence is not yet sufficient for administering PMRT. That is, clinicians should ask themselves: “Would I recommend PMRT for this patient if she had undergone simultaneous ALND, and there were no additional nodal metastases in the nonsentinel nodes?” If the answer is no, ALND should be performed. This discussion should ideally occur before surgery, especially because this could guide patient decision making about reconstruction choices if reconstruction is desired.

### Clinical Question 3

Is PMRT indicated in patients with clinical stage I or II cancers who have received NAST?

#### Updated Recommendation

Patients with axillary nodal involvement that persists after NAST (eg, less than a complete pathologic response) should receive PMRT. Observational data suggest a low risk of locoregional recurrence for patients who have clinically negative nodes and receive NAST or who have a complete pathologic response in the lymph nodes with NAST. However, there is currently insufficient evidence to recommend whether PMRT should be administered or can be routinely omitted in these groups. The panel recommends entering eligible patients in clinical trials that examine this question (type: informal consensus; evidence quality: low; strength of recommendation: weak).

#### Literature Review and Analysis

Neoadjuvant chemotherapy was initially limited to patients with unresectable locally advanced disease to allow mastectomy to be performed. Such patients then generally received PMRT. Whether PMRT is indicated in women with resectable early-stage breast cancer who have received neoadjuvant chemotherapy or endocrine therapy is an issue of increasing importance. There are few studies of the risks of LRF in such patients in relation to either pre- or post-treatment clinical and pathologic features.^66^–^69^ The interpretation of these data are further complicated by the varying use of axillary ultrasound and biopsy before initiation of systemic therapy to enhance detection of clinically positive nodes before NAST and by the downstaging of nodal status by NAST (ie, a complete pathologic response in an individual with pre-NAST positive nodes that were detected on imaging only). The influence of potential risk factors for LRF may be different in patients undergoing NAST and those undergoing surgery before systemic therapy.

The panel agrees that, on the basis of currently available data, patients with persistently involved nodes on ALND after NAST have a sufficiently high risk of LRF to recommend that they receive PMRT, although there are as yet no data from randomized trials showing the effect of such treatment on long-term breast cancer mortality rates. Rates of LRF in patients with residual invasive cancer in the breast but negative axillary nodes after NAST are inconsistent across studies. Although patients with no residual disease in either the breast or axillary nodes seem to have low rates of LRF, there are insufficient data to exclude the possibility that certain subgroups of these patients may still benefit from PMRT (eg, those who had biopsy-proven axillary nodal involvement before chemotherapy or those with tumors with aggressive biologic features). Hence, the panel did not believe that recommendations for or against the use of PMRT could be made with confidence for these latter two groups at this time. The panel recommends entering these patients in clinical trials if available. There are currently two ongoing major multicenter trials in North America for patients with biopsy-proven axillary node involvement before NAST. The NRG Oncology Group 9353 trial, which opened in August 2013, randomly allocates patients with positive axillary fine-needle aspiration cytology or core biopsy before chemotherapy who undergo mastectomy or breast-conserving surgery and have negative nodes on ALND or SNB to either no irradiation or PMRT including the chest wall or reconstructed breast and RNI (if they undergo mastectomy) or breast irradiation or breast plus RNI (if they undergo breast-conserving surgery; ClinicalTrials.gov identifier NCT01872975). The Alliance for Clinical Trials in Oncology A011202 trial addresses another question relevant to patients undergoing NAST, namely, whether patients with a positive SNB after chemotherapy have a different outcome when treated with ALND or axillary radiation therapy without additional surgery (ClinicalTrials.gov identifier NCT01901094). Unfortunately, there are no such trials for patients without biopsy-proven nodal involvement before NAST who are found to have pathologically negative axillary nodes after NAST.

### Clinical Question 4

Should RNI include both the IMNs and supraclavicular-axillary apical nodes when PMRT is used in patients with T1-2 tumors with one to three positive axillary nodes?

#### Updated Recommendation

The panel recommends treatment generally be administered to both the IMNs and the supraclavicular-axillary apical nodes in addition to the chest wall or reconstructed breast when PMRT is used for patients with positive axillary lymph nodes. There may be subgroups that will experience limited, if any, benefits from treating both these nodal areas compared with treating only one or perhaps treating only the chest wall or reconstructed breast. There is insufficient evidence at this time to define such subgroups in detail. Additional research is needed to identify them (type: informal consensus; evidence quality: intermediate; strength of recommendation: moderate).

#### Literature Review and Analysis

The panel agreed the critical question to address in this update should be whether to administer PMRT or not and not to focus on issues of what areas should be treated or how to deliver treatment. However, the panel also deemed it necessary to discuss the issue of RNI in patients with one to three positive nodes in view of the recent publications of the French, Canadian, and European prospective randomized trials and a large Danish retrospective study on this topic.^25^–^27^,^70^

The minimum mandatory target volumes for PMRT that were agreed upon by the panel are the chest wall and supraclavicular-axillary apical nodes in current practice. There remains controversy over when the IMNs and level I and II axillary nodes should be deliberately included. The radiation fields in 20 of the 22 trials in the EBCTCG meta-analysis showing benefit with PMRT included the IMNs, usually with additional regional nodes.^5^

Two randomized trials, conducted by the Canadian Cancer Trials Group (previously the National Cancer Institute of Canada) and the EORTC evaluated the addition of irradiation of the supraclavicular nodes, axillary apical nodes, and IMNs to whole-breast irradiation after breast-conserving surgery (both trials) or after chest wall or no chest wall irradiation after mastectomy (in the EORTC trial only).^26^,^27^ A randomized trial conducted in France addressed the question of the addition of IMN irradiation to chest wall, supraclavicular, and axillary apical nodal irradiation.^25^ All three trials included patients with node-positive and node-negative breast cancers. Finally, a nonrandomized study from Denmark compared results in node-positive patients with right-sided cancers who, according to Danish national guidelines, were to receive IMN irradiation in addition to the breast or chest wall and supraclavicular-infraclavicular nodes with results in patients with left-sided cancers, who were not to undergo IMN irradiation.^70^ Overall findings of these four studies are summarized in Table 3. All found 1 %–5 % reductions in rates of relapse and breast cancer-specific and overall mortalities in patients receiving more extensive irradiation. Some of these differences were statistically significant (eg, overall survival in the EORTC and Danish studies).

**Table 3:** Outcome in Studies of Nodal Irradiation

Study	SFRO^25^	EORTC^27^	NCIC^26^	Danish^70^
Dates of accrual	1991–1997	1996–2004	2000–2007	2003–2007
No. of patients	1,332	4,004	1,832	3,089
Median follow-up (years)	8.6	10.9	9.5	8.9
Irradiated sites	Chest wall + SC-IC ± IMN	Breast and chest wall ± SC-IC-IMNs	Breast ± SC-IC-IMNs	Breast and chest wall + SC-IC ± IMNs
Disease-free survival, %	50 % and 53 %	69 % and 72 %	77 % and 82 %	NR
Distant disease-free survival	NR	75 % and 78 %	83 % and 87 %	70 % and 73 %
Breast cancer-specific mortality	NR	14 % and 12 %	12 % and 10 %	23 % and 21 %
Overall survival	59 % and 63 %	81 % and 82 %	91 % and 92 %	72 % and 76 %

NOTE. Results given first for more limited irradiation and then for more extensive irradiation. All results for the Danish study given at 8 years; all others given at 10 years

Abbreviations: EORTC, European Organisation for Research and Treatment of Cancer; IC, infraclavicular or axillary apex; IMN, internal mammary node; NCIC, National Cancer Institute of Canada; NR, not reported; SC, supraclavicular; SFRO, Société Francaise de Radiation Oncologique

Together, these studies support the effectiveness of RNI. However, their interpretation is complicated by differences in their exact design and in their detailed findings. For example, the French trial included only patients treated with mastectomy; the Canadian trial included only those undergoing breast-conserving surgery; and the EORTC trial mainly included patients treated with breast-conserving surgery, but 24 % of patients were treated with mastectomy. All three randomized trials included patients with negative axillary nodes, but the proportions in each trial were different (25 %, 10 %, and 44 % in the French, Canadian, and EORTC trials, respectively). Any patient with negative nodes with a central- or inner-quadrant primary was eligible for the French and EORTC trials, whereas only node-negative patients with high-risk features were eligible for the Canadian trial (tumor ≥5 cm, tumor ≥2 cm with ≤10 axillary nodes removed, estrogen receptor (ER) negative, histologic grade 3, or lymphovascular invasion present). In the EORTC trial, patients who had undergone mastectomy underwent RNI versus no RNI according to random allocation; chest wall irradiation was administered at the discretion of the treating physicians. The use of systemic therapy and radiation field guidelines and techniques also varied. For example, the IMNs in the first five intercostal spaces were included in the French trial, the first three intercostal spaces in the Canadian trial, and the first three intercostal spaces in the EORTC trial, except for patients with lower inner-quadrant tumors, in whom the first five intercostal spaces could be included. Finally, all patients in the French trial underwent supraclavicular-infraclavicular nodal irradiation, with IMN irradiation being randomly assigned. However, the MA.20 and EORTC trials assigned patients to irradiation of both the supraclavicular-infraclavicular area and IMN nodes or to no nodal irradiation, so the effects of treating these two sites could not be separately evaluated.

Mindful of the limitations of subgroup analyses, reporting of results also differed substantially among these trials, particularly with regard to plausible prognostic or predictive factors. The French trial did not report results for patients with one to three positive nodes separately from those with more involved nodes. Crude rates of any breast cancer event were reduced from 20 % to 16 % in the MA.20 trial and from 33 % to 30 % in the EORTC trial with the addition of RNI, with respective reductions in overall death rates of 1 % and 2 %. However, there was no difference in overall survival at 8 years (83 %) between the two groups in the Danish study (subgroup treatment interactions were not reported). Results in relation to receptor status were reported only for the MA.20 trial. Ten-year disease-free survival rates in the control and RNI arms for patients with ER-positive tumors were 79 % and 81 %, respectively, which were not significantly different; 10-year overall survival rates were 84 % in both arms. However, for patients with ER-negative tumors, 10-year disease-free survival rates were significantly different in the control and RNI arms (71 % and 82 %, respectively); 10-year overall survival rates were 74 % and 81 %, respectively. The EORTC trial found no difference in death rates between the control and RNI arms when chemotherapy alone was used (28 % and 30 %, respectively) or in patients not receiving systemic therapy (14 % and 13 %, respectively). However, although there was no significant difference in mortality with the addition of RNI for patients receiving endocrine therapy alone (a reduction from 21 % to 18 %), there was a statistically significant reduction in patients receiving both endocrine therapy and chemotherapy (from 20 % to 15 %). It is not yet clear whether these different results in different subgroups resulted from chance alone or reflect important clinical distinctions.

The consensus of the panel, on the basis of the EBCTCG meta-analysis and the Canadian and EORTC RNI trials, is that both the IMN and supraclavicular-axillary apical areas should generally be treated when PMRT is used. However, certain subgroups may experience limited benefit from such treatment, and as noted, treating the supraclavicular and IMN areas can result in additional toxicities, with pulmonary and cardiac morbidities being particular concerns even with improved radiotherapy techniques. Additional analyses of these trials and other studies are needed to determine which patients should undergo irradiation of only one or neither of these areas.

In general, the full axilla is not irradiated in those who have had ALND, because recurrence in the dissected axilla is rare, and its inclusion may further increase toxicities, particularly lymphedema.^71^ However, there are circumstances where full axillary irradiation may be considered, such as when ALND is not performed or after ALND in cases with extensive bulky involvement. There are insufficient data to propose recommendations in this area at present.

## Discussion

The panel recommends strongly that input from all clinicians as well as the patient is needed to yield the best results from PMRT. This is best achieved through discussion among providers early in the patient’s treatment course (before or soon after surgery and before or soon after the initiation of systemic therapy), either in the context of a formal tumor board or by referral to the surgical, medical, and radiation oncologists caring for the patient. Patients vary in how much they wish to participate in decision making, but ultimately, their values determine whether the potential long-term benefits of PMRT are sufficient to outweigh potential short- and long-term risks of adverse effects.

Additional information, including a Data Supplement, a Methodology Supplement, evidence tables, and clinical tools and resources are, in part, published with this article and can all be found at www.asco.org/pmrt-guideline and www.asco.org/guidelineswiki. Patient information is available there and at www.cancer.net. Visit www.asco.org/guidelineswiki to provide comments on the guideline or to submit new evidence.

## Electronic supplementary material

Below is the link to the electronic supplementary material.

Supplementary material 1 (DOCX 245 kb)

Supplementary material 2 (DOCX 105 kb)
