# Supplementary material for: Postmastectomy Radiotherapy: An American Society of Clinical Oncology, American Society for Radiation Oncology, and Society of Surgical Oncology Focused Guideline Update
Source: Ann Surg Oncol. 2016 Sep 19;24(1):38–51. doi: 10.1245/s10434-016-5558-8 (PMC5179596; doi:10.1245/s10434-016-5558-8)
Supplement: Supplementary file 1 — Supplementary material 1 (DOCX 245 kb) [file 10434_2016_5558_MOESM1_ESM.docx]

**
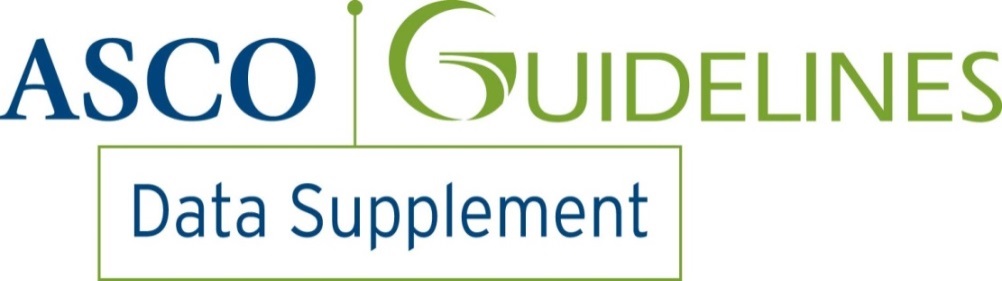
**

**Postmastectomy Radiotherapy: An American Society of Clinical Oncology, American Society for Radiation Oncology, Society of Surgical Oncology Focused Guideline Update**

Table of Contents :

Data Supplement 1: Search Strategy Strings and Dates

- Search A -- Update of Cancer Care Ontario literature search for guideline on locoregional therapy for locally advanced breast cancer
- Search B -- PMRT in women who have received neoadjuvant chemotherapy
- Search C -- Technical aspects of PMRT
- Search D -- Single-center and multi-institutional prospective and retrospective studies of patients treated since the PMRT trials in the EBCTCG meta-analysis were completed

Data Supplement 2: QUOROM Diagrams for Searches A-D

Data Supplement 3: Potential Risk Factors for Local-Regional Recurrence In Patients Treated with Initial Mastectomy and Axillary Dissection

Data Supplement 4: Local-Regional Failure Rates in Patients Undergoing Neoadjuvant Systemic Therapy

**Data Supplement 1: Search Strategy Strings and Dates**

**Search A: Update of Cancer Care Ontario literature search for guideline on locoregional therapy for locally advance breast cancer**

Dates of search: January of 2014 to July of 2015

Limits: The search was restricted to articles published in English, and to systematic reviews, meta-analyses, randomized controlled trials, and practice guidelines

Databases searched: MEDLINE and EMBASE

Search Strategy:

1. (clinical trial or randomized controlled trial).pt. or exp clinical trial/ or random allocation.mp. or random allocation/ or random:.tw. or double-blind method.mp. or double-blind method/ or single-blind method.mp. or single-blind method/ or placebos/ or placebo:.tw.

2. meta-analysis.mp. or meta-analysis/ or meta-analysis.pt. or (meta-analy: or metaanaly: or meta analy:).tw. or (systematic review or systematic overview).mp. or (cochrane or medline or embase or cancerlit).ti. or (hand search or hand-search or manual search).ti. or practice guideline$.mp. or Practice Guideline/ or practice guideline.pt. or practice parameter:.tw.

3. exp Breast Neoplasms/ or exp breast tumor/ or exp breast cancer/ or breast cancer.mp. or breast neoplasm:.mp. or ((cancer: or neoplasm: or tumo?r: or carcinom:) and (breast or mammar:)).mp.

4. exp Radiotherapy Planning, Computer-Assisted/ or exp Radiotherapy, Computer-Assisted/ or exp Radiotherapy/ or (radiotherapy or radiation treatment or radiation therapy or irradiation treatment or irradiation therapy).mp.

5. (1 or 2) and 3 and 4

6. limit 5 to yr="2014-current"

7. 5 and (201312: or 2014: or 2015:).ed.

8. 5 and (201312: or 2014: or 2015::).dd.

9. 6 or 7 or 8

10. 9 not (editorial or letter or comment).pt

**Data Supplement 1: Search Strategy Strings and Dates**

**Search B -- PMRT in women who have received neoadjuvant chemotherapy**

Dates of search: 1999 to 2015

Limits: The search was restricted to articles published in English, and to systematic reviews, meta-analyses, and randomized controlled trials.

Databases searched: PUBMED

Search Strategy:

(("English"[Language] AND 1999[PDAT] : 2015[PDAT]) NOT ("animals"[MeSH Terms] NOT "humans"[MeSH Terms]) NOT clinical trial, phase I[ptyp] NOT "pediatrics"[MeSH Terms]) AND ("Breast Neoplasms"[mesh] OR (breast[tiab] AND (cancer[tiab] OR cancers[tiab] OR neoplasm[tiab] OR malignacy[tiab] OR malignacies[tiab]))) AND ("neoplasm recurrence, local"[MeSH Terms] OR ((locoregional[tiab] OR local[tiab] OR loco[tiab]) AND (recurrence[tiab] OR relapse[tiab] OR Failure[tiab] OR survival[tiab]))) AND (("1-3"[tiab] OR "one to three"[tiab] OR "1 to 3"[tiab] OR "node positive"[tiab] OR N1[tiab] OR "N1-3"[tiab]) AND (node[tiab] OR nodes[tiab])) AND ("radiotherapy"[Subheading] OR "radiotherapy"[All Fields] OR "radiotherapy"[MeSH Terms]) AND (("mastectomy, simple"[MeSH Terms] OR ("mastectomy"[All Fields] AND "simple"[All Fields]) OR "simple mastectomy"[All Fields] OR "mastectomy"[All Fields] OR "mastectomy"[MeSH Terms]) OR postmastectomy[tiab] OR PMRT[tiab] OR (("1-3"[tiab] OR "one to three"[tiab] OR "1 to 3"[tiab]) AND (node[tiab] OR nodes[tiab]))) AND (neoadjuvant[ti] OR "neoadjuvant therapy"[MeSH Terms])

**Data Supplement 1: Search Strategy Strings and Dates**

**Search C -- Lymphatic Irradiation and PMRT**

Dates of search: 1999 to 2015

Limits: The search was restricted to articles published in English, and to systematic reviews, meta-analyses, and randomized controlled trials.

Databases searched: PUBMED

Search Strategy:

(("English"[Language] AND 1999[PDAT] : 2015[PDAT]) NOT ("animals"[MeSH Terms] NOT "humans"[MeSH Terms]) NOT clinical trial, phase I[ptyp] NOT "pediatrics"[MeSH Terms]) AND ("Breast Neoplasms"[mesh] OR (breast[tiab] AND (cancer[tiab] OR cancers[tiab] OR neoplasm[tiab] OR malignacy[tiab] OR malignacies[tiab]))) AND ("neoplasm recurrence, local"[MeSH Terms] OR ((locoregional[tiab] OR local[tiab] OR loco[tiab]) AND (recurrence[tiab] OR relapse[tiab] OR Failure[tiab] OR survival[tiab]))) AND (("1-3"[tiab] OR "one to three"[tiab] OR "1 to 3"[tiab] OR "node positive"[tiab] OR N1[tiab] OR "N1-3"[tiab]) AND (node[tiab] OR nodes[tiab])) AND ("radiotherapy"[Subheading] OR "radiotherapy"[All Fields] OR "radiotherapy"[MeSH Terms]) AND (("mastectomy, simple"[MeSH Terms] OR ("mastectomy"[All Fields] AND "simple"[All Fields]) OR "simple mastectomy"[All Fields] OR "mastectomy"[All Fields] OR "mastectomy"[MeSH Terms]) OR postmastectomy[tiab] OR PMRT[tiab] OR (("1-3"[tiab] OR "one to three"[tiab] OR "1 to 3"[tiab]) AND (node[tiab] OR nodes[tiab]))) AND ("lymphatic irradiation"[MeSH Terms] OR ("lymphatic"[All Fields] AND "irradiation"[All Fields]) OR "lymphatic irradiation"[All Fields])

**Data Supplement 1: Search Strategy Strings and Dates**

**Search D -- Single-center and multi-institutional prospective and retrospective studies of patients treated since the PMRT trials in the EBCTCG meta-analysis were completed**

Dates of search: 1999 to July 2015

Limits: The search was restricted to articles published in English, retrospective or prospective study published between January 2001-July 2015; patients accrued from 1985 or after; 150 or more patients explicitly identified with T1-2 cancers with 1-3 positive nodes; patients were not treated with neoadjuvant chemotherapy; and median follow-up 48 months or longer.

Databases searched: PUBMED

Search Strategy:

#1 (Eng[lang] AND 1999:2015[dp]) NOT(animals[mesh] NOT humans[mesh]) NOT (clinical trial, phase I

[ptyp]) NOT (pediatrics [mesh] )

#2 ("Breast Neoplasms"[mesh] OR (breast[tiab] AND (cancer[tiab] OR cancers[tiab] OR neoplasm[tiab]

OR malignacy[tiab] OR malignacies[tiab])))

#3 ((Neoplasm Recurrence, Local[MESH]) OR (( locoregional[tiab] OR local[tiab] OR loco[tiab]) AND

(recurrence[tiab] OR relapse[tiab] OR Failure[tiab] OR survival[tiab])))

#4 (("1-3"[tiab] OR "one to three"[tiab] OR "1 to 3"[tiab] OR "node positive"[tiab] OR N1[tiab] OR

"N1-3"[tiab]) AND (node[tiab] OR nodes[tiab]))

#5 (radiotherapy[all fields])

#6 (mastectomy[all fields] OR postmastectomy[tiab] OR PMRT[tiab] OR (( "1-3"[tiab] OR "one to three" [tiab] OR "1 to 3"[tiab] ) AND (node[tiab] OR nodes[tiab] ) ))

#7 (neoadjuvant[ti] OR (Neoadjuvant Therapy)

Then: #1 AND #2 AND #3 AND #4 AND #5 AND #6 NOT #7

**DATA SUPPLEMENT 2*.* QUOROM Diagrams**

QUOROM diagram for Search A, update of Cancer Care Ontario literature search for guideline on locoregional therapy for locally advanced breast cancer

The updated literature search yielded 1294 records

From the 1294 records, 7 potentially relevant abstracts identified.

Klepin HD; Pitcher BN; Ballman KV; Kornblith AB; Hurria A; Winer EP; Hudis C; Cohen HJ; Muss HB; Kimmick GG.

Ovid MEDLINE(R) In-Process &amp; Other Non-Indexed Citations Journal of oncology practice/American Society of Clinical Oncology. 10(5):e285-92, 2014 Sep. potentially relevant abstracts identified

None of the publications provided new evidence that would warrant substantive modification of the practice recommendations as drafted.

One of the publications informed the ASCO Panel’s comments, and is referenced in the manuscript.

**DATA SUPPLEMENT 2*.* QUOROM Diagrams**

QUOROM diagram for Search B, PMRT in women who have received neoadjuvant chemotherapy

The updated literature search yielded 15 records

From the records, 0 potentially relevant abstracts identified

Klepin HD; Pitcher BN; Ballman KV; Kornblith AB; Hurria A; Winer EP; Hudis C; Cohen HJ; Muss HB; Kimmick GG.

Ovid MEDLINE(R) In-Process &amp; Other Non-Indexed Citations Journal of oncology practice/American Society of Clinical Oncology. 10(5):e285-92, 2014 Sep. potentially relevant abstracts identified

None of the publications provided new evidence that would warrant substantive modification of the practice recommendations

None of the publications from the literature search informed the ASCO Panel’s comments.

**DATA SUPPLEMENT 2*.* QUOROM Diagrams**

QUOROM diagram for Search C, Technical aspects of PMRT

The updated literature search yielded 23 records

From the records, 1 potentially relevant abstract identified

Klepin HD; Pitcher BN; Ballman KV; Kornblith AB; Hurria A; Winer EP; Hudis C; Cohen HJ; Muss HB; Kimmick GG.

Ovid MEDLINE(R) In-Process &amp; Other Non-Indexed Citations Journal of oncology practice/American Society of Clinical Oncology. 10(5):e285-92, 2014 Sep. potentially relevant abstracts identified

One of the publications from the formal search and two other publications identified from panel members’ files provided new evidence that informed the practice recommendations

No additional publications from the literature search informed the ASCO Panel’s comments.

**DATA SUPPLEMENT 2*.* QUOROM Diagrams**

QUOROM diagram for Search D, Single-center and multi-institutional prospective and retrospective studies of patients treated since the PMRT trials in the EBCTCG meta-analysis were completed

The updated literature search yielded 214 records

From the 214 records, 25 potentially relevant abstracts identified.

Klepin HD; Pitcher BN; Ballman KV; Kornblith AB; Hurria A; Winer EP; Hudis C; Cohen HJ; Muss HB; Kimmick GG.

Ovid MEDLINE(R) In-Process &amp; Other Non-Indexed Citations Journal of oncology practice/American Society of Clinical Oncology. 10(5):e285-92, 2014 Sep. potentially relevant abstracts identified

After review of the full-text articles,17 of the publications provided evidence that informed the practice recommendations as drafted.

**Data Supplement 3: Potential Risk Factors for Local-Regional Recurrence In Patients Treated with Initial Mastectomy and Axillary Dissection**

Patient Age

Few studies have examined how narrow ranges of patient age affect the risk of local-regional failure (LRF) in patients with pT1-2N1 cancers treated with mastectomy and axillary lymph node dissection (ALND) who receive systemic therapy without postmastectomy radiation therapy (PMRT). A study from the Eastern Cooperative Oncology Group (ECOG) found that patients age 34 years or younger had a trend towards higher LRF rates than older patients, while patients older than 65 years had lower failure rates in both patients with pT1 and pT2 cancers (Table S1).^1^ A study of more recently treated patients from the M.D. Anderson Cancer Center (MDACC) found that patients younger than age 40 also had a higher LRF rate than older patients, but the absolute differences were smaller, and the 10 patients older than 70 years had a higher failure rate than other patients (Table S2).^2^ Several studies using a single division to define "younger" and "older" patients also suggest that patient age 40 years or younger is a clinically important risk factor for LRF (Table S3).^3-6^

Tumor Size and Multicentricity

Tumor stage had very little impact of the risk of LRF in several multi-institutional studies,^1,7,8^ though two studies from the MDACC suggested it had a larger one, with 10-year LRF rates of 9% and 26% in the T1 and T2 subgroups in an earlier study and 2% and 10%, respectively, in a later one.^9,10^ Tumor size may have different impacts on different subgroups. For example, in the International Breast Cancer Study Group (IBCSG) study, T-stage was a statistically significant risk factor on multivariate analysis for postmenopausal patients, but not for premenopausal ones.^7^

Few studies have looked at smaller divisions of tumor size for patients with 1-3 positive nodes.^1,9^ The study from the MDACC found that patients with tumors smaller than 1 cm had a substantially lower risk of LRF than patients with larger cancers, but that tumor size had little effect otherwise (Table S4).^9^

The issue of whether multicentricity or multifocality increases the risk of LRF after mastectomy was first raised in a study from the University of Pennsylvania and Fox Chase Cancer Center by Fowble and colleagues published in 1993.^11^ However, there have been few more recent studies of this factor. A study of patients treated at MDACC from 1975-1994 found gross multicentric disease (defined as tumor involving more than one quadrant or separated by 4 cm of more that was clinically apparent or defined on gross pathologic examination) was a risk factor for the series as a whole.^12^ However, there were only 23 patients with 1-3 nodes who had gross multicentric disease of 466 total. Ten-year rates of LRF with and without gross multicentric disease were 17% and 13%, respectively, which was not significantly different with a median follow-up of 116 months in the entire series. The most recent study examining this, which included only patients with pN1 disease treated at MSKCC from 1995-2006, found multicentric or multifocal disease (not defined) in 58% of their patients who did not receive PMRT.^13^ Rates of LRF were not reported in relation to this variable, but it was not statistically significant on either univariate or multivariate analysis.

Number of Involved Nodes and Nodal Ratio

Studies examining LRF in relation to the exact number of involved nodes have had inconsistent findings as to how this affects the risk of LRF (Table S5).^10,13,14^ The proportion of recovered nodes involved by cancer (or “nodal ratio”) has been a much more consistent predictor, with higher rates when the nodal ratio is greater than 15-25% (Table S6).^3,5,6,15^ However, other factors may modify this risk sufficiently so that nodal ratio alone cannot be used for decision making. For example, a combined study of patients with 1-3 positive axillary nodes from the MDACC (462 patients) and British Columbia Cancer Agency (BCCA) (82 patients) found that a nodal ratio of 0.20 or lower was associated with a significantly smaller risk of LRF in the combined study population.^16^ However, the 10-year LRF rate for “low-risk” patients treated at the MDACC was 11%, compared to 18% for the BCCA patients; for “high-risk” patients, the respective rates were 23% and 28%. (Note that nodal ratio is not applicable to patients who have sentinel lymph node biopsy only.)

Size of Nodal Metastases and Extracapsular Extension

Few studies have examined the effect of the size of nodal metastases on LRF (Table S7).^10,13,17^ Each used a different dividing point between "smaller" and "larger" tumor deposits, which reflects differences in how institutions report tumor size within nodes. Two of these studies suggested nodal metastasis size had very limited impact, while the effect was substantial in one study. None of these studies examined the interaction of metastasis size with other factors, such as the exact number of involved nodes or tumor size.

The presence of extracapsular extension did not increase the risk of LRF in patients with 1-3 involved nodes in several older series,^18-21^ although in two studies extracapsular extension substantially increased the risk of chest-wall recurrence.^22,23^ However, it is not clear whether systemic therapy was routinely used in these patients. More recent studies in which systemic therapy was clearly given have had mixed findings (Table S8).^9,10,13,15^ Having any extracapsular extension substantially increased the LRF risk in a series from the Cleveland Clinic^15^ and in a study from the MDACC when extranodal extension of 2 mm or greater was present, compared to none or less than 2 mm extension.^9^ However, this was not found in a more recent study from MDACC^10^ or one from Memorial Sloan-Kettering Cancer Center (MSKCC).^13^ Central pathology review was not performed in these studies, making the findings perhaps less reliable.

Margin Width or Status

There are no studies examining the impact of margin status for only patients with 1-3 positive nodes. Several studies in patients with either negative or both negative and positive axillary nodes have generally, but not always, suggested there are increased LRF rates in patients with "close" or positive margins, compared to wider ones (Table S9).^12,24,25^ However, it is not clear whether there was a standardized approach to assessing the deep margin status in these series. The magnitude of the effect of margin status varies between the series, and they contain small numbers of patients. It also seems likely that margin status has a significant impact on the risk of LRF when combined with other factors, but not by itself. A study from the BCCA of patients with negative nodes with positive margins found that LRF rates were low except when patients had one or more additional risk factors (Table S10).^26^. Similarly, in the study from Brigham and Women's/Faulkner Hospital, Boston in whom 83% of patients had negative nodes, the crude LRF rate was 3% (1/39) in patients with a positive margin without lymphovascular invasion (LVI), compared to 27% (4/15) when both LVI and a positive margin were found.^25^

Lymphovascular Invasion

The presence of LVI substantially increased the risk of LRF in most, but not all, recent series examining this issue (Table S11).^2,3,5,6,13,27,28^ However, there are substantial problems in interpreting and using these data. First, interobserver variability between pathologists reduces the reliability of this diagnosis, although the use of stringent criteria can result in concordance rates in excess of 80%.^29^ Second, only some of these studies examined the effect of LVI in patient subgroups defined according to other prognostic factors. In the IBCSG study, the presence of LVI was an important risk factor for premenopausal patients but not postmenopausal patients on multivariate analysis.^7^ In an older MDACC study, LVI was significantly associated with the risk of LRF for patients with 4 or more positive nodes, but not 1-3 positive nodes; however, exact rates in patients with 1-3 positive nodes were not reported.^12^

Histologic Grade

Studies of patients with pT1-2N1 cancer have differed on whether or by how much high histologic grade increases the risk of LRF, compared to that in patients with grade 1 or 2 cancers (Table S12).^3,6,7,13,15,28,30^ None of these studies subdivided the results further in relation to hormone receptor or HER-2 status, which tend to be more often adverse in high-grade tumors.

Biomarkers and Genomic Factors

The prognostic value of estrogen receptor (ER) expression for LRF after mastectomy and ALND has varied substantially in different series of patients with pT1-2N1 cancers routinely receiving systemic therapy (Table S13).^1,3,6,8,13^ The ER status was a statistically significant predictor of LRF on multivariate analysis in the ECOG study^1^, but there was no difference in rates according to ER status in an analysis of the combined Danish PMRT trials including patients of any nodal status.^31^ However, having negative progesterone receptor (PR) status was a statistically significant risk factor in the latter study.

There are few data on the role of HER-2 expression in determining the risk of LRF (Table S14).^6,31,32^ Two of these did not show a substantially higher risk in patients with HER-2 positive cancers receiving chemotherapy regimens that did not contain trastuzumab than in HER-2 negative patients, while one did. However, only the latter study was restricted to patients with 1-3 positive nodes.^6^ There as yet few data on whether anti-HER2 therapies reduce rates of LRF in patients undergoing mastectomy without PMRT.^33^

Combinations of these receptor biomarkers may or may not be of more importance than individual ones. With a median follow-up of 204 months, the Danish study showed that the 78 patients with “triple negative” tumors (negative ER and PR and normal expression of HER-2) had a crude LRF rate of 32%, compared to 33% in the 432 patients with other subgroups.^31^ A study from the European Institute of Oncology in Milan found crude LRF rates of 11% (of 121 patients) and 7% (of 1621 patients) in these subgroups, respectively, at median follow-up of 74 months.^34^ However, both studies contained patients with both positive and negative axillary nodes. A study from Beijing of 319 patients with T1-2N1 tumors treated from 2000-2004 who did not undergo PMRT found, with a median follow-up of 47 months for the entire study population, that the actuarial 5-year LRF rate for patients with triple negative receptor status was 12%, compared to 17% for patients with HER-2 positive tumors but negative ER and PR, 18% for patients with positive ER or PR and positive HER-2, and 9% for patients with positive ER or PR and negative HER-2.^35^ The number of patients in each subgroup was not reported.

There are as yet few data on how gene-expression analysis might be used to predict the risk of LRF after mastectomy for patients with 1-3 involved axillary nodes. A study of the 21-gene recurrence score in patients with negative axillary nodes treated with mastectomy suggested a high score was prognostic of an increased risk of LRF.^36^ However, this assay did not substantially change 10-year LRF rates in a study of patients with ER or PR positive tumors with 1-3 positive nodes treated in the NSABP B-28 trial, which were lower than 10% in all recurrence score groups.^37^

Combinations of Risk Factors

Several investigators have proposed using combinations of features to better assess the risk of LRF in unirradiated patients with pT1-2N1 cancers. A study from the BCCA of 821 patients treated from 1989-1997 with median follow-up of 92 months found age 45 or younger, nodal ratio greater than 25%, medial tumor location, and ER negative receptor status were significant variable on recursive partition analysis.^3^ Patients could be separated into groups whose LRF rate at 10 years was as low as 8% or as high as 51%. A study from Ankara, Turkey of 326 patients accrued from 1990-2004 with median follow-up time of 70 months found age 35 or younger, the presence of LVI, and nodal ratio greater than 15% were significant risk factors.^5^ The 10-year LRF in the 300 patients with no or one risk factor was 4%, compared to 23% for the 26 patients with two or three risk factors. A study from MSKCC of 924 patients treated from 1995-2006 found age 50 and younger and the presence of LVI were significant risk factors on multivariate analysis.^13^ With median follow-up of 84 months, the 5-year LRF rate in patients older than age 50 without LVI was 1%, compared to 10% in those age 50 or younger with LVI. (The number of patients in these subgroups was not reported). A study of 271 patients treated at the Cleveland Clinic from 2000-2007 found that extracapsular extension and histologic grade 3 were significant risk factors, with median follow-up 56 months.^15^ Patient with zero (139), one (116), or two (16) risk factors had 5-year LRF rates of 4%, 8%, and 50%, respectively. Finally, an analysis of 368 patients treated from 2001-2005 in Tianjin, China found that age 40 or younger, tumor size larger than 3 cm, the presence of LVI, and ER negative receptor status were significant risk factors for LRF.^6^ With median follow-up of 86 months, the 8-year LRF rate for 322 patients with 0-2 risk factors was 7%, compared to 37% for the 46 patients with 3-4 risk factors. However, none of these schemes have yet been validated in series of patients from other institutions.

**Data Supplement 4: Local-Regional Failure Rates in Patients Undergoing Neoadjuvant Systemic Therapy**

There are no data yet from randomized trials on the role of PMRT in patients with clinical Stage I or II tumors who undergo neoadjuvant systemic therapy (NAST). The first important data on this subject came from the MDACC (Table S15).^38^ With a median follow-up time of 4.1 years in patients treated from 1974-1998, the 5-year risk of LRF varied according to the initial clinical stage and pathologic axillary status following neoadjuvant chemotherapy (NACT). (They scored patients who suffered LRF following distant failure as having a LRF, as well as patients with LRF as a first site of failure. Most investigators have not included such patients in their analyses.) Tamoxifen use was associated with a dramatically lower risk of LRF; however, its impact was not assessed or reported within each prognostically-relevant subgroup, and hence (despite their multivariate analysis) it is not clear that this effect is not related to the differential distribution of prognostic factors between patients who did or did not receive tamoxifen, rather than to a treatment effect. An expansion of the MDACC series to include 132 clinical Stage I-II patients treated from 1974-2001 found that, with a median follow-up of 46 months, the 5-year actuarial risk of LRF for 62 patients with both clinically and pathologically-negative nodes was 5%; the rate for 42 patients with initial clinical T1-2 lesions with 1-3 positive axillary nodes at surgery was also 5% (crude rate, 2/42); and the rate for the 6 patients with 4 or more positive nodes was 67%.^39^ For 19 patients with clinical T3N0 tumors, the rate was 29%. Patients with a pathologic complete response had a crude LRF of 4% (1/25). This group also reported results in 226 patients without inflammatory breast cancer treated from 1982-2002 who had a pathologic complete response after NACT who then either had or did not have PMRT.^40^ With a median follow-up time of 62 months in surviving patients, none of the 22 unirradiated patients or 10 irradiated patients with initial clinical Stage I-II disease had a LRF. Finally, a study concentrating on patients with cT3N0 tumors found that, with median follow-up of 75 months, the risk of LRF in unirradiated was related to both pathologic response and tumor nuclear grade (Table S16).^41^ The use of PMRT substantially reduced failure rates.

The largest fully published experience in this area is that from a combined analysis of the NSABP B-18 and B-27 trials (Table S17).^42^ This study found that both clinical stage and pathologic response were important prognostic features for LRF, with median follow-up of 11.75 years.

Finally, a presentation at the 2015 annual meeting of the American Society of Clinical Oncology examined LRF rates after NACT in 1001 patients treated with (851) or without (150) PMRT on three trials of NACT conducted in Germany with median follow-up of 50 months (Table S18).^43^

In summary, all studies have found that patients with pathologic axillary node involvement following NACT had substantially higher LRF failure rates than patients with negative nodes, and patients with pathologic complete response had lower rates than patients with negative axillary nodes but residual invasive cancer in the breast. However, it is very difficult to compare results between these studies due to their different ways of subgrouping patients both before and after NAST, the small number of patients in subgroups, and varied lengths of follow-up. When comparable subgroup results are reported, their results have sometimes been discordant. For example, the 10-year LRF rate in patients with cT3N0 tumors with residual disease in the breast but negative axillary nodes in the NSABP study was 10%,^42^ compared to 14% at 5 years in the MDACC study.^41^

Table S1. Age and T-Stage Combined and 10-Year Local-Regional Failure Rates After Mastectomy and Chemotherapy Without Radiation Therapy for Patients with 1-3 Positive Axillary Nodes: Eastern Cooperative Oncology Group, 1978-1987.

| Age (years) | Local-Regional Failure: T1 | Local-Regional Failure: T2 |
| --- | --- | --- |
| <34 | 21% (24) | 18% (45) |
| 35-44 | 13% (93) | 11% (156) |
| 45-54 | 15% (132) | 12% (161) |
| 55-64 | 9% (108) | 14% (142) |
| >65 | 6% (50) | 8% (72) |

Number of patients in subgroup in parentheses. Median follow-up without recurrence, 12.1 years.

Data from: Recht A, Gray R, Davidson NE, et al: Locoregional failure ten years after mastectomy and adjuvant chemotherapy with or without tamoxifen without irradiation: experience of the Eastern Cooperative Oncology Group. J Clin Oncol 17:1689-1700, 1999.

Table S2. Age and 10-Year Local-Regional Failure Rates After Mastectomy and Chemotherapy Without Radiation Therapy for Patients with T1-2 Cancers with 1-3 Positive Axillary Nodes in Relation to Age: M.D. Anderson Cancer Center, 2000-2007.

| Age | Number of Patients | Local-Regional Failure |
| --- | --- | --- |
| <40 | 59 | 9% |
| 41-50 | 122 | 4% |
| 51-60 | 131 | 2% |
| 61-70 | 63 | 3% |
| >71 | 10 | 17% |

Data from: McBride A, Allen P, Woodward W, et al: Locoregional recurrence risk for patients with T1,2 breast cancer with 1-3 positive lymph nodes treated with mastectomy and systemic treatment. Int J Radiat Oncol Biol Phys 89:392-8, 2014.

Table S3. Age and Local-Regional Failure Rates After Mastectomy and Chemotherapy Without Radiation Therapy for Patients with T1-2 Cancers with 1-3 Positive Axillary Nodes in Studies Using Single Age Divisions.

| Series | Dates of Accrual | Follow-Up (months) | Definition of "Younger" | Measure | LRF: Younger Patients | LRF: Older Patients |
| --- | --- | --- | --- | --- | --- | --- |
| BCCA ^3^ | 1989-1997 | 92 | <39 | 10-year actuarial | 24% (52) | 12% (769) |
| Taipei ^4^ | 1991-2005 | 40 | <40 | Crude | 20% (66) | 8% (317) |
| Ankara ^5^ | 1990-2004 | 70 | <34 | Hazard ratio | 6.8 (39/287) | |
| Tianjin ^6^ | 2001-2005 | 86 | <39 | 8-year actuarial | 17% (78) | 9% (290) |

Number of patients in subgroup in parentheses.

Abbreviations:

BCCA: British Columbia Cancer Agency.

HR: hazard ratio.

LRF: local-regional failure.

Table S4. Tumor Size and 10-Year Total Local-Regional Failure Rates After Mastectomy and Chemotherapy Without Radiation Therapy for Patients with 1-3 Positive Axillary Nodes: M.D. Anderson Cancer Center, 1975-1994.

| Size (cm) | Number of Patients | Local-Regional Failure |
| --- | --- | --- |
| <1 | 36 | 3% |
| 1.1-2.0 | 154 | 11% |
| 2.1-3.0 | 120 | 15% |
| 3.1-4.0 | 69 | 15% |
| 4.1-5.0 | 25 | 16% |

Median follow-up, 116 months.

Data from: Katz A, Strom EA, Buchholz TA, et al: Locoregional recurrence patterns after mastectomy and doxorubicin-based chemotherapy: implications for postoperative irradiation. J Clin Oncol 18:2817-2827, 2000.

Table S5. Number of Involved Nodes and Local-Regional Failure Rates After Mastectomy and Chemotherapy Without Radiation Therapy for Patients with T1-2 Cancers with 1-3 Positive Axillary Nodes.

| Series | Dates of Accrual | Follow-Up (months) | Measure | LRF: 1 Node | LRF: 2 Nodes | LRF: 3 Nodes |
| --- | --- | --- | --- | --- | --- | --- |
| BCCA ^14^ | 1989-1997 | 90 | 10-year actuarial | 12% (326) | 27% (147) | 24% (69) |
| MSKCC ^13^ | 1995-2006 | 84 | 5-year actuarial | 3% (577) | 6% (242) | 7% (105) |
| MDACC ^10^ | 1997-2002 | 90 | 10-year actuarial | 3% (176) | 8% (69) | 0 (21) |
| Tianjin ^6^ | 2001-2005 | 86 | 8-year actuarial | 10% (292) | | 15%(76) |

Number of patients in subgroups in parentheses.

Abbreviations:

BCCA: British Columbia Cancer Agency.

LRF: local-regional failure.

MDACC: M.D. Anderson Cancer Center.

MSKCC: Memorial Sloan-Kettering Cancer Center.

Note: Patients with 1 or 2 positive nodes grouped together in the study from Tianjin.

Table S6. Nodal Ratio and Local-Regional Failure Rates After Mastectomy and Chemotherapy Without Radiation Therapy for Patients with T1-2 Cancers with 1-3 Positive Axillary Nodes.

| Series | | Dates of Accrual | | Follow-Up (months) | | Definition of "Lower" | | Measure | | LRF: Lower Ratio | LRF: Higher Ratio |
| --- | --- | --- | --- | --- | --- | --- | --- | --- | --- | --- | --- |
| BCCA ^3^ | | 1989-1997 | | 92 | | <25% | | 10-year actuarial | | 10% (674) | 25% (141) |
| Ankara ^5^ | 1990-2004 | | 70 | | <15% | | Hazard ratio | | 13.0 (326) | | |
| Cleveland ^15^ | 2000-2007 | | 62 | | <25% | | Hazard ratio | | 2.7 (271) | | |
| Tianjin ^6^ | | 2001-2005 | | 86 | | <20% | | 8-year actuarial | | 10% (335) | 19% (33) |

Number of patients in subgroups in parentheses.

Abbreviations:

BCCA: British Columbia Cancer Agency.

LRF: local-regional failure.

Table S7. Size of Nodal Metastases and Local-Regional Failure Rates After Mastectomy and Chemotherapy Without Radiation Therapy for Patients with T1-2 Cancers with 1-3 Positive Axillary Nodes.

| Series | Dates of Accrual | Follow-Up (months) | Definition of "Smaller" | Measure | LRF: Smaller | LRF: Larger |
| --- | --- | --- | --- | --- | --- | --- |
| MSKCC ^13^ | 1995-2006 | 84 | <2 mm | 5-year actuarial | 2% (259) | 5% (661) |
| Moffitt, Tampa ^17^ | 1996-2007 | 66 | <5 mm | Crude | 7% (149) | 20% (55) |
| MDACC ^10^ | 1997-2002 | 90 | <10 mm | 10-year actuarial | 4% (195) | 6% (39) |

Number of patients in subgroups in parentheses.

Abbreviations:

LRF: local-regional failure.

MDACC: M.D. Anderson Cancer Center.

MSKCC: Memorial Sloan-Kettering Cancer Center.

Table S8. Presence of Extracapsular Extension and Local-Regional Failure Rates After Mastectomy and Chemotherapy Without Radiation Therapy for Patients with T1-2 Cancers with 1-3 Positive Axillary Nodes.

| Series | Dates of Accrual | Follow-Up (months) | Measure | LRF: None | LRF: Present |
| --- | --- | --- | --- | --- | --- |
| MDACC ^9^ | 1975-1994 | 116 | Crude | 10% (332) | 28% (36) |
| MSKCC ^13^ | 1995-2006 | 84 | 5-year actuarial | 4% (809) | 4% (115) |
| MDACC ^10^ | 1997-2002 | 90 | Crude | 9% (225) | 5% (41) |
| Cleveland Clinic ^15^ | 2000-2007 | 62 | Hazard ratio | 4.3 (224/47) | |

Number of patients in subgroups in parentheses.

Note: Extracapsular extension scored only if 2 mm or greater in the MDACC series. Patients with unknown extent of extracapsular extension are excluded.

Abbreviations:

LRF: local-regional failure.

MDACC: M.D. Anderson Cancer Center.

MSKCC: Memorial Sloan-Kettering Cancer Center.

Table S9. Margin Width and Crude Local-Regional Failure Rates After Mastectomy Without Radiation Therapy for Patients with T1-2 Cancers with Negative or Positive Axillary Nodes.

| Series | Dates of Accrual | Follow-Up (months) | Definition of "Close" (mm) | LRF: Positive | LRF: Close | LRF: Wider |
| --- | --- | --- | --- | --- | --- | --- |
| MDACC ^12^ | 1975-1994 | 116 | <5 mm | 41% (29) | | 16% (965) |
| Fox Chase Cancer Center ^24^ | 1985-1994 | 59 | <2 mm | 50% (2) | 18% (17) | --- |
| Brigham & Women's/Faulkner Hospital, Boston ^25^ | 1998-2005 | 80 | <2 mm | 9% (54) | 3% (68) | 3% (275) |

Abbreviations:

LRF: local-regional failure.

MDACC: M.D. Anderson Cancer Center.

Table S10. Crude Local-Regional Failure Rates in Patients with Positive Margins After Mastectomy Without Radiation Therapy for Patients with T1-2 Cancers with Negative Axillary Nodes: British Columbia Cancer Agency, 1989-1998.

| Factor | LRF: Absent | LRF: Present |
| --- | --- | --- |
| Age <50 years | 6% (2/33) | 20% (4/20) |
| T2 | 4% (1/27) | 19% (5/26) |
| Grade 3 | 9% (3/35) | 23% (3/13) |
| Lymphovascular Invasion | 11% (4/38) | 17% (2/12) |
| Systemic Therapy Given | 16% (4/25) | 7% (2/28) |

Median follow-up, 92 months.

Abbreviations:

LRF: local-regional failure.

Data from: Truong PT, Olivotto IA, Speers CH, et al: A positive margin is not always an indication for radiotherapy after mastectomy in early breast cancer. Int J Radiat Oncol Biol Phys 58:797-804, 2004.

Table S11. Presence of Lymphovascular Invasion and Local-Regional Failure Rates After Mastectomy and Chemotherapy Without Radiation Therapy for Patients with T1-2 Cancers with 1-3 Positive Axillary Nodes.

| Series | Dates of Accrual | Follow-Up (months) | Measure | LRF: No LVI | LRF: LVI Present |
| --- | --- | --- | --- | --- | --- |
| BCCA ^3^ | 1989-1997 | 92 | 10-year actuarial | 10% (370) | 13% (386) |
| Ankara ^5^ | 1990-2004 | 70 | Hazard ratio | 3.3 (262/64) | |
| Tokyo ^27^ | 1990-2000 | 112 | Hazard ratio | Limited: 1.7 (?)  Extensive: 3.1 (?) | |
| Shikoku ^28^ | 1990-2002 | 82 | 8-year actuarial | 3% (197) | 5% (51) |
| MSKCC ^13^ | 1995-2006 | 84 | 5-year actuarial | 2% (516) | 8% (408) |
| MDACC ^2^ | 2000-2007 | 84 | 10-year actuarial | 3% (260) | 6% (124) |
| Tianjin ^6^ | 2001-2005 | 86 | 8-year actuarial | 8% (296) | 23% (72) |

Number of patients in subgroups in parentheses.

Notes: The Tokyo series contained 1086 patients with 1-3 positive nodes, but the numbers of patients with and without LVI were not reported for this subgroup. "Extensive LVI" was defined as 10 or more areas of LVI found. The Shikoku series distinguishes lymphatic invasion from blood vessel invasion; results for lymphatic invasion given here.

Abbreviations:

BCCA: British Columbia Cancer Agency.

LRF: local-regional failure.

LVI: lymphovascular invasion.

MDACC: M.D. Anderson Cancer Center.

MSKCC: Memorial Sloan-Kettering Cancer Center.

Table S12. Histologic Grade and Local-Regional Failure Rates After Mastectomy and Chemotherapy Without Radiation Therapy for Patients with T1-2 Cancers with 1-3 Positive Axillary Nodes.

| Series | Dates of Accrual | Follow-Up (months) | Measure | LRF: Grade 1-2 | LRF: Grade 3 |
| --- | --- | --- | --- | --- | --- |
| IBCSG ^7^ | 1978-1993 | 174 | 10-year actuarial | 17% (650) | 23% (353) |
| BCCA ^3^ | 1989-1997 | 92 | 10-year actuarial | 9% (434) | 18% (322) |
| MGH ^30^ | 1990-2004 | 84 | Crude | 3% (96) | 15% (59) |
| Shikoku ^28^ | 1990-2002 | 82 | 8-year actuarial | 4% (103) | 5% (145) |
| MSKCC ^13^ | 1995-2006 | 84 | Crude | 2% (204) | 6% (572) |
| Cleveland Clinic ^15^ | 2000-2007 | 62 | Hazard ratio | 3.6 (157/101) | |
| Tianjin ^6^ | 2001-2005 | 86 | 8-year actuarial | 10% (298) | 13% (70) |

Notes: The Shikoku series includes patients with unknown grade with the grade 1-2 patients, Crude failure rate in the MSKCC series were nil of 20 patients with grade 1 cancers and 2% for 204 patients with grade 2 cancers.

Abbreviations:

BCCA: British Columbia Cancer Agency.

IBCSG: International Breast Cancer Study Group.

LRF: local-regional failure.

MDACC: M.D. Anderson Cancer Center.

MGH: Massachusetts General Hospital.

MSKCC: Memorial Sloan-Kettering Cancer Center.

Table S13. Estrogen Receptor Status and Local-Regional Failure Rates After Mastectomy and Chemotherapy Without Radiation Therapy for Patients with T1-2 Cancers with 1-3 Positive Axillary Nodes.

| Series | Dates of Accrual | Follow-Up (months) | Measure | LRF: ER Negative | LRF: ER Positive |
| --- | --- | --- | --- | --- | --- |
| ECOG ^1^ | 1978-1987 | 145 | 10-year actuarial | T1: 17% (124) | 11% (283) |
|  |  |  |  | T2: 13% (210) | 12% (366) |
| NSABP ^8^ | 1984-1994 | 133 | 10-year actuarial | Age <49: 19% (390) | 19% (448) |
|  |  |  |  | Age >50: 12% (210) | 10% (385) |
| BCCA ^3^ | 1989-1997 | 92 | 10-year actuarial | 19% (175) | 10% (568) |
| MSKCC ^13^ | 1995-2006 | 84 | 5-year actuarial | 7% (197) | 4% (710) |
| Tianjin ^6^ | 2001-2005 | 86 | 8-year actuarial | 16% (178) | 6% (190) |

Notes: The effect of ER was reported only for T2 lesions in the NSABP series.

Abbreviations:

BCCA: British Columbia Cancer Agency.

ECOG: Eastern Cooperative Oncology Group.

ER: estrogen receptor status.

LRF: local-regional failure.

MSKCC: Memorial Sloan-Kettering Cancer Center.

NSABP: National Surgical Adjuvant Breast and Bowel Program.

Table S14. HER-2 Receptor Status and Local-Regional Failure Rates After Mastectomy and Chemotherapy Without Radiation Therapy for Patients with T1-2 Cancers with Negative or Positive Axillary Nodes.

| Series | Dates of Accrual | Follow-Up (months) | Measure | LRF: HER-2 Negative | LRF: HER-2 Positive |
| --- | --- | --- | --- | --- | --- |
| DBCG ^31^ | 1982-1990 | 204 | 15-year actuarial | 32% (404) | 39% (106) |
| BCCA (pN0) ^32^ | 1986-1992 | 154 | 10-year actuarial | 13% (802) | 9% (104) |
| Tianjin (pN1) ^6^ | 2001-2005 | 86 | 8-year actuarial | 8% (238) | 20% (77) |

Abbreviations:

BCCA: British Columbia Cancer Agency.

DBCG: Danish Breast Cancer Group.

LRF: local-regional failure.

Table S15. Five-Year Local-Regional Failure Rates After Mastectomy and Neoadjuvant Chemotherapy Without Radiation Therapy: M.D. Anderson Cancer Center, 1974-1988.

| Clinical T, yp N | Local-Regional Failure Rate |
| --- | --- |
| cT1-2, ypN0 | 5% (1/19) |
| cT1-2, ypN+ | 13% (4/42) |
| cT3-4, ypN0 | 34% (6/23) |
| cT3-4, ypN+ | 36% (4/64) |

Number of failures and patients in subgroups in parentheses. Median follow-up, 4.1 years.

Abbreviations:

ypN0: pathologically negative axillary nodes after neoadjuvant chemotherapy.

ypN+: pathologically positive axillary nodes after neoadjuvant chemotherapy.

Data from: Buchholz TA, Tucker SL, Masullo L, et al: Predictors of local-regional recurrence after neoadjuvant chemotherapy and mastectomy without radiotherapy. J Clin Oncol 20:17-23, 2002.

Table S16. Five-Year Local-Regional Failure Rates After Neoadjuvant Chemotherapy and Mastectomy Without or With Radiation Therapy for Patients with Initial Clinical Stage T3N0: M.D. Anderson Cancer Center 1985-2004.

| Group | No PMRT | PMRT |
| --- | --- | --- |
| All Patients | 24% (43) | 4% (119) |
| pCR | 0 (4) | 0 (9) |
| ypN0 | 14% (32) | 2% (62) |
| ypN+ | 53% (11) | 5% (57) |
| Nuclear grade 1-2 | 13% (17) | 2% (63) |
| Nuclear grade 3 | 37% (19) | 10% (51) |

Number of patients in subgroups within parentheses. Median follow-up, 75 months.

Abbreviations:

pCR: pathologic complete response following neoadjuvant chemotherapy.

PMRT: postmastectomy radiation therapy.

ypN0: pathologically negative axillary nodes following neoadjuvant chemotherapy.

ypN+: pathologically positive axillary nodes following neoadjuvant chemotherapy.

Data from: Nagar H, Mittendorf EA, Strom EA, et al: Local-regional recurrence with and without radiation therapy after neoadjuvant chemotherapy and mastectomy for clinically staged T3N0 breast cancer. Int J Radiat Oncol Biol Phys 81:782-7, 2011.

Table S17. Initial Clinical Stage, Pathologic Response After Neoadjuvant Chemotherapy, and Ten-Year Local-Regional Failure Rates After Mastectomy Without Radiation Therapy: National Surgical Adjuvant Breast and Bowel Project B-18 and B-27 Trials, 1998-2000.

| Initial Clinical Stage | Pathologic Response Following Chemotherapy | | |
| --- | --- | --- | --- |
|  | No Disease in Breast or Axillary Nodes | Residual Breast Disease, Negative Axillary Nodes | Positive Axillary Nodes |
| T1-2N0 | 6.5% (46) | 6.3% (178) | 12.2% (184) |
| T1-2N1 | 0 (21) | 10.8% (37) | 17% (143) |
| T3N0 | 6.2% (16) | 9.8% (95) | 14.6% (179) |
| T3N1 | 0 (11) | 9.2% (84) | 22.4% (128) |

Median follow-up was 11.75 years in the combined data-set. Number of patients in subgroups in parentheses.

Data from: Mamounas EP, Anderson SJ, Dignam JJ, et al: Predictors of locoregional recurrence after neoadjuvant chemotherapy: results from combined analysis of National Surgical Adjuvant Breast and Bowel Project B-18 and B-27. J Clin Oncol 30:3960-6, 2012.

Table S18. Five-Year Local-Regional Failure Rates After Neoadjuvant Chemotherapy and Mastectomy Without or With Radiation Therapy: Gepar Trials.

| Group | No PMRT | PMRT |
| --- | --- | --- |
| pCR | 11% (20) | 8% (81) |
| No pCR | 19% (130) | 16% (770) |
| cT1-2 | 11% (98) | 12% (343) |
| cT3-4 | 31% (51) | 17% (445) |
| cN0 | 11% (74) | 10% (270) |
| cN+ | 26% (75) | 17% (574) |
| ypN0 | 16% (100) | 9% (314) |
| ypN+ | 20% (45) | 19% (517) |
| cN+->ypN0 | 24% (40) | 10% (178) |

Median follow-up, 50 months. Number of patients in subgroups in parentheses.

Abbreviations:

pCR: pathologic complete response following neoadjuvant chemotherapy.

PMRT: postmastectomy radiation therapy.

cN0: clinically negative axillary nodes prior to chemotherapy.

cN+: clinically positive axillary nodes prior to chemotherapy.

ypN0: pathologically negative axillary nodes following neoadjuvant chemotherapy.

ypN+: pathologically positive axillary nodes following neoadjuvant chemotherapy.

Data presented at the American Society of Clinical Oncology Annual Meeting, Chicago IL, June 2015. See: Krug D, Lederer B, Debus J, et al: Relationship of omission of adjuvant radiotherapy to outcomes of locoregional control and disease-free survival in patients with or without pCR after neoadjuvant chemotherapy for breast cancer: A meta-analysis on 3481 patients from the Gepar-trials. J Clin Oncol 33 (suppl):abstr 1008, 2015.

**References**

1. Recht A, Gray R, Davidson NE, et al: Locoregional failure ten years after mastectomy and adjuvant chemotherapy with or without tamoxifen without irradiation: experience of the Eastern Cooperative Oncology Group. J Clin Oncol 17:1689-1700, 1999

2. McBride A, Allen P, Woodward W, et al: Locoregional recurrence risk for patients with T1,2 breast cancer with 1-3 positive lymph nodes treated with mastectomy and systemic treatment. Int J Radiat Oncol Biol Phys 89:392-8, 2014

3. Truong PT, Olivotto IA, Kader HA, et al: Selecting breast cancer patients with T1-T2 tumors and one to three positive axillary nodes at high postmastectomy locoregional recurrence risk for adjuvant radiotherapy. Int J Radiat Oncol Biol Phys 61:1337-1347, 2005

4. Yang PS, Chen CM, Liu MC, et al: Radiotherapy can decrease locoregional recurrence and increase survival in mastectomy patients with T1 to T2 breast cancer and one to three positive nodes with negative estrogen receptor and positive lymphovascular invasion status. Int J Radiat Oncol Biol Phys 77:516-22, 2010

5. Yildirim E, Berberoglu U: Local recurrence in breast carcinoma patients with T(1-2) and 1-3 positive nodes: indications for radiotherapy. Eur J Surg Oncol 33:28-32, 2007

6. Lu C, Xu H, Chen X, et al: Irradiation after surgery for breast cancer patients with primary tumours and one to three positive axillary lymph nodes: yes or no? Curr Oncol 20:e585-92, 2013

7. Wallgren A, Bonetti M, Gelber RD, et al: Risk factors for locoregional recurrence among breast cancer patients: results from International Breast Cancer Study Group Trials I through VII. J Clin Oncol 21:1205-1213, 2003

8. Taghian A, Jeong JH, Mamounas E, et al: Patterns of locoregional failure in patients with operable breast cancer treated by mastectomy and adjuvant chemotherapy with or without tamoxifen and without radiotherapy: results from five National Surgical Adjuvant Breast and Bowel Project randomized clinical trials. J Clin Oncol 22:4247-4254, 2004

9. Katz A, Strom EA, Buchholz TA, et al: Locoregional recurrence patterns after mastectomy and doxorubicin-based chemotherapy: implications for postoperative irradiation. J Clin Oncol 18:2817-2827, 2000

10. Sharma R, Bedrosian I, Lucci A, et al: Present-day locoregional control in patients with T1 or T2 breast cancer with 0 and 1 to 3 positive lymph nodes after mastectomy without radiotherapy. Ann Surg Oncol 17:2899-908, 2010

11. Fowble B, Yeh I-T, Schultz DJ, et al: The role of mastectomy in patients with Stage I-II breast cancer presenting with gross multifocal or multicentric disease or diffuse microcalcifications. Int J Radiat Oncol Biol Phys 27:567-573, 1993

12. Katz A, Strom EA, Buchholz TA, et al: The influence of pathologic tumor characteristics on locoregional recurrence rates following mastectomy. Int J Radiat Oncol Biol Phys 50:735-742, 2001

13. Moo TA, McMillan R, Lee M, et al: Selection criteria for postmastectomy radiotherapy in T1-T2 tumors with 1 to 3 positive lymph nodes. Ann Surg Oncol 20:3169-74, 2013

14. Truong PT, Berthelet E, Lee J, et al: The prognostic significance of the percentage of positive/dissected axillary lymph nodes in breast cancer recurrence and survival in patients with one to three positive axillary lymph nodes. Cancer 103:2006-2014, 2005

15. Tendulkar RD, Rehman S, Shukla ME, et al: Impact of postmastectomy radiation on locoregional recurrence in breast cancer patients with 1-3 positive lymph nodes treated with modern systemic therapy. Int J Radiat Oncol Biol Phys 83:e577-81, 2012

16. Truong PT, Woodward WA, Thames HD, et al: The ratio of positive to excised nodes identifies high-risk subsets and reduces inter-institutional differences in locoregional recurrence risk estimates in breast cancer patients with 1-3 positive nodes: an analysis of prospective data from British Columbia and the M. D. Anderson Cancer Center. Int J Radiat Oncol Biol Phys 68:59-65, 2007

17. Harris EE, Freilich J, Lin HY, et al: The impact of the size of nodal metastases on recurrence risk in breast cancer patients with 1-3 positive axillary nodes after mastectomy. Int J Radiat Oncol Biol Phys 85:609-14, 2013

18. Donegan WL, Stine SB, Samter TG: Implications of extracapsular nodal metastases for treatment and prognosis of breast cancer. Cancer 72:778-782, 1993

19. Perera F, Fisher BJ, Cooke A, et al: Locoregional recurrence and extranodal extension in patients receiving systemic therapy for axillary node positive breast cancer (Abstr.). Int J Radiat Oncol Biol Phys 27(suppl. 1):265, 1993

20. Pierce LJ, Oberman HA, Strawderman MH, et al: Is an axillary boost necessary in node positive Stage II disease in the presence of microscopic extracapsular extension? (Abstr.). Breast Cancer Res Treat 27:152, 1993

21. Leonard CE, Corkill M, Tompkin J, et al: Are axillary recurrence and overall survival affected by axillary extranodal tumor extension in breast cancer? Implications for radiation therapy. J Clin Oncol 13:47-53, 1995

22. Kuske RR, Sanchez M, Farr GH, et al: Extracapsular axillary nodal extension (ECE) in breast cancer: patterns of recurrence, arm edema, and survival after mastectomy with and without irradiation (Abstr.). Int J Radiat Oncol Biol Phys 45 (suppl.):157, 1999

23. Cheng JC, Chen CM, Liu MC, et al: Locoregional failure of postmastectomy patients with 1-3 positive axillary lymph nodes without adjuvant radiotherapy. Int J Radiat Oncol Biol Phys 52:980-988, 2002

24. Freedman GM, Fowble BL, Hanlon AL, et al: A close or positive margin after mastectomy is not an indication for chest wall irradiation except in women aged fifty or younger. Int J Radiat Oncol Biol Phys 41:599-605, 1998

25. Childs SK, Chen YH, Duggan MM, et al: Surgical margins and the risk of local-regional recurrence after mastectomy without radiation therapy. Int J Radiat Oncol Biol Phys 84:1133-8, 2012

26. Truong PT, Olivotto IA, Speers CH, et al: A positive margin is not always an indication for radiotherapy after mastectomy in early breast cancer. Int J Radiat Oncol Biol Phys 58:797-804, 2004

27. Matsunuma R, Oguchi M, Fujikane T, et al: Influence of lymphatic invasion on locoregional recurrence following mastectomy: indication for postmastectomy radiotherapy for breast cancer patients with one to three positive nodes. Int J Radiat Oncol Biol Phys 83:845-52, 2012

28. Hamamoto Y, Ohsumi S, Aogi K, et al: Are there high-risk subgroups for isolated locoregional failure in patients who had T1/2 breast cancer with one to three positive lymph nodes and received mastectomy without radiotherapy? Breast Cancer 21:177-82, 2014

29. Pinder SE, Ellis IO, Galea M, et al: Pathological prognostic factors in breast cancer. III. Vascular invasion: relationship with recurrence and survival in a large study with long-term follow-up. Histopathology 24:41-7, 1994

30. MacDonald SM, Abi-Raad RF, Alm El-Din MA, et al: Chest wall radiotherapy: middle ground for treatment of patients with one to three positive lymph nodes after mastectomy. Int J Radiat Oncol Biol Phys 75:1297-303, 2009

31. Kyndi M, Sorensen FB, Knudsen H, et al: Estrogen receptor, progesterone receptor, HER-2, and response to postmastectomy radiotherapy in high-risk breast cancer: the Danish Breast Cancer Cooperative Group. J Clin Oncol 26:1419-26, 2008

32. Kwan W, Al-Tourah AJ, Speers C, et al: Does HER2 status influence locoregional failure rates in breast cancer patients treated with mastectomy for pT1-2pN0 disease? Ann Oncol 21:988-93, 2010

33. Lanning RM, Morrow M, Riaz N, et al: The effect of adjuvant trastuzumab on locoregional recurrence of human epidermal growth factor receptor 2-positive breast cancer treated with mastectomy. Ann Surg Oncol 22:2517-25, 2015

34. Kneubil MC, Brollo J, Botteri E, et al: Breast cancer subtype approximations and loco-regional recurrence after immediate breast reconstruction. Eur J Surg Oncol 39:260-5, 2013

35. Wang SL, Li YX, Song YW, et al: Triple-negative or HER2-positive status predicts higher rates of locoregional recurrence in node-positive breast cancer patients after mastectomy. Int J Radiat Oncol Biol Phys 80:1095-101, 2011

36. Mamounas EP, Tang G, Fisher B, et al: Association between the 21-gene recurrence score assay and risk of locoregional recurrence in node-negative, estrogen receptor-positive breast cancer: results from NSABP B-14 and NSABP B-20. J Clin Oncol 28:1677-83, 2010

37. Mamounas EP, Tang G, Paik S, et al: The 21-gene Recurrence Score (RS) predicts risk of loco-regional recurrence (LRR) in node (+), ER (+) breast cancer (BC) after adjuvant chemotherapy and tamoxifen: results from NSABP B-28. Ann Surg Oncol 20 (1 suppl):S6 (abstr 2), 2013

38. Buchholz TA, Tucker SL, Masullo L, et al: Predictors of local-regional recurrence after neoadjuvant chemotherapy and mastectomy without radiotherapy. J Clin Oncol 20:17-23, 2002

39. Garg AK, Strom EA, McNeese MD, et al: T3 disease at presentation or pathologic involvement of four or more lymph nodes predict for locoregional recurrence in stage II breast cancer treated with neoadjuvant chemotherapy and mastectomy without radiotherapy. Int J Radiat Oncol Biol Phys 59:138-145, 2004

40. McGuire SE, Gonzalez-Angulo AM, Huang EH, et al: Postmastectomy radiation improves the outcome of patients with locally advanced breast cancer who achieve a pathologic complete response to neoadjuvant chemotherapy. Int J Radiat Oncol Biol Phys 68:1004-9, 2007

41. Nagar H, Mittendorf EA, Strom EA, et al: Local-regional recurrence with and without radiation therapy after neoadjuvant chemotherapy and mastectomy for clinically staged T3N0 breast cancer. Int J Radiat Oncol Biol Phys 81:782-7, 2011

42. Mamounas EP, Anderson SJ, Dignam JJ, et al: Predictors of locoregional recurrence after neoadjuvant chemotherapy: results from combined analysis of National Surgical Adjuvant Breast and Bowel Project B-18 and B-27. J Clin Oncol 30:3960-6, 2012

43. Krug D, Lederer B, Debus J, et al: Relationship of omission of adjuvant radiotherapy to outcomes of locoregional control and disease-free survival in patients with or without pCR after neoadjuvant chemotherapy for breast cancer: A meta-analysis on 3481 patients from the Gepar-trials. J Clin Oncol 33 (suppl):abstr 1008, 2015
