# Supplementary material for: Postmastectomy Radiotherapy: An American Society of Clinical Oncology, American Society for Radiation Oncology, and Society of Surgical Oncology Focused Guideline Update
Source: Ann Surg Oncol. 2016 Sep 19;24(1):38–51. doi: 10.1245/s10434-016-5558-8 (PMC5179596; doi:10.1245/s10434-016-5558-8)
Supplement: Supplementary file 2 — Supplementary material 2 (DOCX 105 kb) [file 10434_2016_5558_MOESM2_ESM.docx]

**
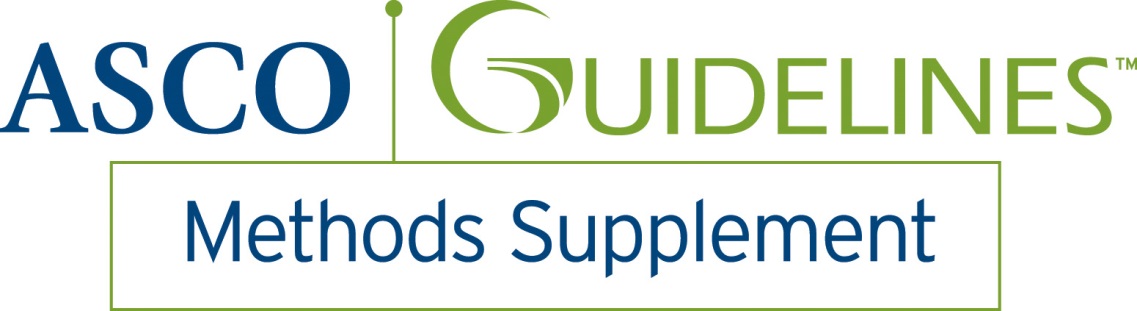
**

**TITLE: Postmastectomy Radiotherapy: An American Society of Clinical Oncology, American Society for Radiation Oncology, Society of Surgical Oncology Focused Guideline Update**

**Table of Contents**

1. **OVERVIEW: GENERAL PRINCIPLES FOR ALL ASCO GUIDELINE PRODUCTS**

Panel Composition

Guideline Development Process

Revision Dates and Methods

Systematic Literature Review

Literature Search Strategy

1. **DEVELOPMENT OF RECOMMENDATIONS**

Guide for Types of Recommendations

Guide for Rating Recommendations and Strength of Evidence

Guide for Rating Strength of Evidence

Guide for Rating Potential for Bias

**1. OVERVIEW: GENERAL PRINCIPLES FOR ALL ASCO GUIDELINE PRODUCTS:**

Practice guidelines are systematically developed statements that assist practitioners and patients in making decisions about care. Attributes of good guidelines include validity, reliability, reproducibility, clinical applicability, flexibility, clarity, multidisciplinary process, review of evidence, and documentation. Guidelines may be useful in producing better care and decreasing cost. Specifically, utilization of clinical guidelines may provide:

1. Improvements in outcomes
2. Improvements in medical practice
3. A means for minimizing inappropriate practice variation
4. Decision support tools for practitioners
5. Points of reference for medical orientation and education
6. Criteria for self-evaluation
7. Indicators and criteria for external quality review
8. Assistance with reimbursement and coverage decisions
9. Criteria for use in credentialing decisions
10. Identification of areas where future research is needed

***Panel Composition***

The ASCO Clinical Practice Guidelines Committee (CPGC) and the ASCO Breast Cancer Guideline Advisory Group (GAG) convened an Expert Panel with multidisciplinary representation in medical oncology, radiation oncology, surgical oncology, and community oncology. The panel included a member of Practice Guidelines Implementation Network, and patient/advocacy representation*.* The Expert Panel was led by two Co-Chairs who had the primary responsibility for the development and timely completion of the guideline. The Panel had one face-to-face meeting and a single webinar. The Co-Chairs and ASCO staff prepared a draft guideline for review and rating by the Expert Panel. The Expert Panel members are listed in Appendix Table A1 (online only).

For this joint ASCO-ASTRO-SSO focused guideline update, ASTRO and SSO each provided two formal representatives. All ASCO guidelines are reviewed and approved by the ASCO Clinical Practice Guidelines Committee. The focused update was also reviewed by ASTRO’s Guidelines Committee and approved by the ASTRO Board of Directors; the update was reviewed by SSO’s Breast Cancer Disease Site Work Group and approved by the SSO Quality Committee and Executive Council.

***Guideline Development Process***

The full Expert Panel met on two occasions and corresponded frequently through email; progress on guideline development was driven primarily by the Co-Chairs and ASCO staff. The purpose of the Panel meetings was for members to contribute content, provide critical review, interpret evidence, and finalize the guideline recommendations based upon the consideration of the evidence. All members of the Expert Panel participated in the preparation of the draft guideline document, which was then disseminated for external review and submitted to the *Journal of Clinical Oncology (JCO)*, the International Journal of Radiation Oncology • Biology • Physics (IJROBP), and Annals of Surgical Oncology the for peer review and publication.

***Revision Dates and Methods***

The Co-Chairs and two Committee members designated by the Co-Chairs will determine the need for guideline updates or revisions based on periodic examination of the literature. When appropriate, that Committee will recommend revised guidelines to the CPGC for review and approval.

The SIGNALS Approach to Guideline Updating

At annual intervals, the Expert Panel Co-Chairs and two Committee members designated by the Co-Chairs will determine the need for revisions to the guideline based on an examination of current literature. If necessary, an Expert Panel will be (re)convened to discuss potential changes. When appropriate, the Expert Panel will recommend revised guidelines to the Clinical Practice Guideline Committee (CPGC) for review and approval.

The ASCO CPCG’s Methods Subcommittee approved use of a “signals” approach to facilitate guideline updating. This approach outlines formal criteria for identifying new, practice-changing data—signals—that might translate into revised practice recommendations.

The threshold for embarking on ASCO guideline updates that translate into new or revised recommendations--the presence of a signal in the literature—is high. Two major categories of changes are recognized as potential signals:

• a potentially invalidating change in evidence: opposing findings, evidence of substantial harm, evidence of a superior new treatment; and

• A major change in evidence: important changes in efficacy but not opposing findings, expansion of treatment such as evidence of efficacy in a new population, important caveat.

• Of note, there can be reasons other than the scientific literature to initiate a guideline update, including regulatory decisions that affect existing practice recommendations and can require rapid, ad hoc updates.

As described in the following steps, the signals approach relies on a combination of literature searching and expert opinion to inform the need for guideline updating.

Step 1: Reviewing the protocol that was used for the previous systematic review.

Expert Panel leadership, in consultation with other content experts if needed, should review the currency and validity of the guideline clinical questions, target population, interventions, and outcomes. If any of these components of the protocol are out-of-date, the protocol should be revised by the Expert Panel leadership prior to the literature review.

Step 2: Completing the formal literature review and seeking Expert Panel input.

Staff members conduct annual literature searches and review the search yield in consultation with the Expert Panel leadership to identify—or to rule out—signals for updating a guideline. The sources to mine for signals in the published literature include (a) PDQ information summaries, (b) PubMed, and (c) the National Guideline Clearinghouse. The results of the literature search and the initial determination regarding the presence or absence of signals are distributed to the Expert Panel for comment. The Co-Chairs/Steering Committee may also choose to survey the Expert Panel regarding the presence or absence of signals (see Figures 1 and 2).

***Systematic Literature Review***

ASCO guidelines are based on systematic reviews of the literature. A protocol for each systematic review defines parameters for a targeted literature search. Additional parameters include relevant study designs, literature sources, types of reports, and pre-specified inclusion and exclusion criteria for literature identified.

***Literature Search Strategy***

The Expert Panel developed its recommendations based on evidence identified through online searches of the MEDLINE, EMBASE, and Pubmed data bases. Details of the searches are provided in the Data Supplement.

**DEVELOPMENT OF RECOMMENDATIONS**

The guideline recommendations were rated, in part, using the principles of the GuideLines Into DEcision Support (GLIDES) methodology.^1^

**Guide for Types of Recommendations**

| **Type of Recommendation** | **Definition** |
| --- | --- |
| **Evidence based** | There was sufficient evidence from published studies to inform a recommendation to guide clinical practice. |
| **Formal consensus** | The available evidence was deemed insufficient to inform a recommendation to guide clinical practice. Therefore, the Expert Panel used a formal consensus process to reach this recommendation, which is considered the best current guidance for practice. The Panel may choose to provide a rating for the strength of the recommendation (i.e., “strong,” “moderate,” or “weak”). The results of the formal consensus process are summarized in the guideline and reported in the Data Supplement. |
| **Informal consensus** | The available evidence was deemed insufficient to inform a recommendation to guide clinical practice. The recommendation is considered the best current guidance for practice, based on informal consensus of the Expert Panel. The Panel agreed that a formal consensus process was not necessary for reasons described in the literature review and discussion. The Panel may choose to provide a rating for the strength of the recommendation (i.e., “strong,” “moderate,” or “weak”). |
| **No**  **recommendation** | There is insufficient evidence, confidence, or agreement to provide a recommendation to guide clinical practice at this time. The Panel deemed the available evidence as insufficient and concluded it was unlikely that a formal consensus process would achieve the level of agreement needed for a recommendation. |

**Guide for Strength of Recommendations**

| **Rating for**  **Strength of Recommendation** | **Definition** |
| --- | --- |
| **Strong** | There is high confidence that the recommendation reflects best practice. This is based on (1) strong evidence for a true net effect (e.g., benefits exceed harms); (2) consistent results, with no or minor exceptions; (3) minor or no concerns about study quality; and/or (4) the extent of panelists’ agreement. Other compelling considerations (discussed in the guideline’s literature review and analyses) may also warrant a strong recommendation. |
| **Moderate** | There is moderate confidence that the recommendation reflects best practice. This is based on (1) good evidence for a true net effect (e.g., benefits exceed harms); (2) consistent results, with minor and/or few exceptions; (3) minor and/or few concerns about study quality; and/or (4) the extent of panelists’ agreement. Other compelling considerations (discussed in the guideline’s literature review and analyses) may also warrant a moderate recommendation. |
| **Weak** | There is some confidence that the recommendation offers the best current guidance for practice. This is based on (1) limited evidence for a true net effect (e.g., benefits exceed harms); (2) consistent results, but with important exceptions; (3) concerns about study quality; and/or (4) the extent of panelists’ agreement. Other considerations (discussed in the guideline’s literature review and analyses) may also warrant a weak recommendation. |

**Guide for Rating Strength of Evidence**

| **Rating for**  **Strength of Evidence** | **Definition** |
| --- | --- |
| **High** | High confidence that the available evidence reflects the true magnitude and direction of the net effect (i.e., balance of benefits *v* harms) and that further research is very unlikely to change either the magnitude or direction of this net effect. |
| **Intermediate** | Moderate confidence that the available evidence reflects the true magnitude and direction of the net effect. Further research is unlikely to alter the direction of the net effect; however, it might alter the magnitude of the net effect. |
| **Low** | Low confidence that the available evidence reflects the true magnitude and direction of the net effect. Further research may change either the magnitude and/or direction this net effect. |
| **Insufficient** | Evidence is insufficient to discern the true magnitude and direction of the net effect. Further research may better inform the topic. The use of the consensus opinion of experts is reasonable to inform outcomes related to the topic. |

**Guide for Rating of Potential for Bias**

| **Rating of Potential for Bias** | **Definitions for Rating Potential for Risk of Bias in Randomized Controlled Trials** |
| --- | --- |
| **Low risk** | No major features in the study that risk biased results, and none of the limitations are thought to decrease the validity of the conclusions. The study avoids problems such as failure to apply true randomization, selection of a population unrepresentative of the target patients, high dropout rates, and no intention-to-treat analysis; and key study features are described clearly (including the population, setting, interventions, comparison groups, measurement of outcomes, and reasons for dropouts). |
| **Intermediate** | The study is susceptible to some bias, but flaws are not sufficient to invalidate the results. Enough of the items introduce some uncertainty about the validity of the conclusions. The study does not meet all the criteria required for a rating of good quality, but no flaw is likely to cause major bias. The study may be missing information, making it difficult to assess limitations and potential problems. |
| **High risk** | There are significant flaws that imply biases of various types that may invalidate the results. Several of the items introduce serious uncertainty about the validity of the conclusions. The study has serious errors in design, analysis, or reporting; large amounts of missing information; or discrepancies in reporting. |

**References**

1. Shiffman RN, Michel G, Rosenfeld RM, et al: Building better guidelines with BRIDGE-Wiz: development and evaluation of a software assistant to promote clarity, transparency, and implementability. J Am Med Inform Assoc 19:94-101, 2012
